# Proteomic Characterization of Grade II Furcation Defects: A Discovery‐Phase Study

**DOI:** 10.1111/jre.70108

**Published:** 2026-04-09

**Authors:** Georgios S. Chatzopoulos, Dimitra Sakellari, Eleftherios G. Kaklamanos, Lazaros Tsalikis

**Affiliations:** ^1^ Department of Preventive Dentistry, Periodontology and Implant Biology, School of Dentistry Aristotle University of Thessaloniki Thessaloniki Greece

## Abstract

This study analyzed the gingival crevicular fluid (GCF) proteome of 35 patients with Grade II furcation defects using the Olink Reveal platform. Comparison with non‐periodontitis sites identified 227 differentially expressed proteins. A machine‐learning pipeline prioritized a 34‐protein signature (including GLIPR1, MME, and IRAG2) that accurately differentiated furcation defects from health (AUC = 0.887), reflecting a distinct profile of severe inflammation and neutrophil activation.

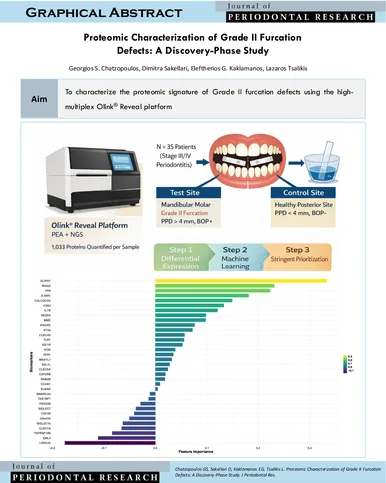

## 1 Introduction

Gingival crevicular fluid (GCF) is an ideal biofluid for monitoring periodontal pathogenesis as its composition reflects the local host‐bacteria interaction [1]. While traditional clinical parameters measure past tissue destruction, proteomic analysis allows for the simultaneous quantification of hundreds of proteins, offering a comprehensive view of the “exudatome” and the complex biological networks driving disease [2].

Targeted high‐throughput proteomics, specifically the Proximity Extension Assay (PEA), enables highly sensitive quantification of protein panels from small sample volumes. Previous studies utilizing such technologies have successfully identified multi‐protein signatures capable of distinguishing periodontal health from disease with high accuracy [3]. However, most research has focused on general periodontitis or active progression [4]. The specific proteomic microenvironment of furcation defects—anatomically complex areas—remains largely unexplored. The unique, sheltered microanatomy of a Grade II furcation creates an isolated ecological niche that fosters biofilm accumulation; this plausibly triggers a distinct, intensified localized host immune response compared to easily accessible interproximal sites.

This study aims to characterize the proteomic signature of Grade II furcation defects using the high‐multiplex Olink Reveal platform. By comparing these advanced lesions to control sites within the same individuals (paired design), it was hypothesized that furcation defects exhibit a unique inflammatory profile distinct from health. This discovery‐phase investigation seeks to identify candidate biomarkers that reflect the specific inflammatory burden of these challenging lesions, providing the essential groundwork for future objective tools to monitor site‐specific risk.

## 2 Methods

This cross‐sectional study was approved by the Ethics Committee of the School of Dentistry at the Aristotle University of Thessaloniki (Protocol No. 155/14‐03‐2022). Thirty‐five systemically healthy patients with Stage III/IV periodontitis were recruited. Participants had completed initial periodontal therapy (Step 1) but presented with residual Grade II furcation defects.

A split‐mouth design was employed. From each patient, GCF was collected from one test site (mandibular molar, Grade II furcation [5], PPD > 4 mm, BOP+) and one control site (posterior site, PPD < 4 mm, BOP‐). GCF was collected using Periopaper strips and analyzed using the Olink Reveal platform (Olink Proteomics, Uppsala, Sweden), quantifying 1033 proteins.

To identify a robust diagnostic signature from the initial 1033 proteins quantified by the Olink Reveal platform, a multistep bioinformatics pipeline was employed. First, differential expression analysis identified candidate proteins (limma). Next, a penalized logistic regression model (Elastic Net) with 5‐fold cross‐validation performed multivariate feature selection. Finally, candidates were subjected to strict prioritization criteria (detection>LOD in 90% of samples and Log2FC>control interquartile range) to distill the initial multiplex panel into a final, robust subset of proteins. Detailed methodology, including inclusion/exclusion criteria and bioinformatics settings, is provided in the Appendix S1.

## 3 Results

The study cohort (mean age 54.7 ± 9.1 years) comprised 20 females and 15 males. Clinical parameters confirmed significant disease severity at the test sites (mean PPD 6.34 ± 1.56 mm) compared to control sites (mean PPD 2.40 ± 0.55 mm; *p* < 0.001) (Table S1).

Principal Component Analysis (PCA) revealed distinct clustering between furcation and control samples. Differential expression analysis identified 227 proteins with significant changes (|Log2FC| > 1, adj. *p* < 0.05), of which 154 were upregulated in furcation defects (Figure 1A). Functional enrichment analysis indicated that these upregulated proteins were heavily involved in cytokine‐cytokine receptor interactions, innate immune responses, and TNF signaling (Figure 1B).

**FIGURE 1:**
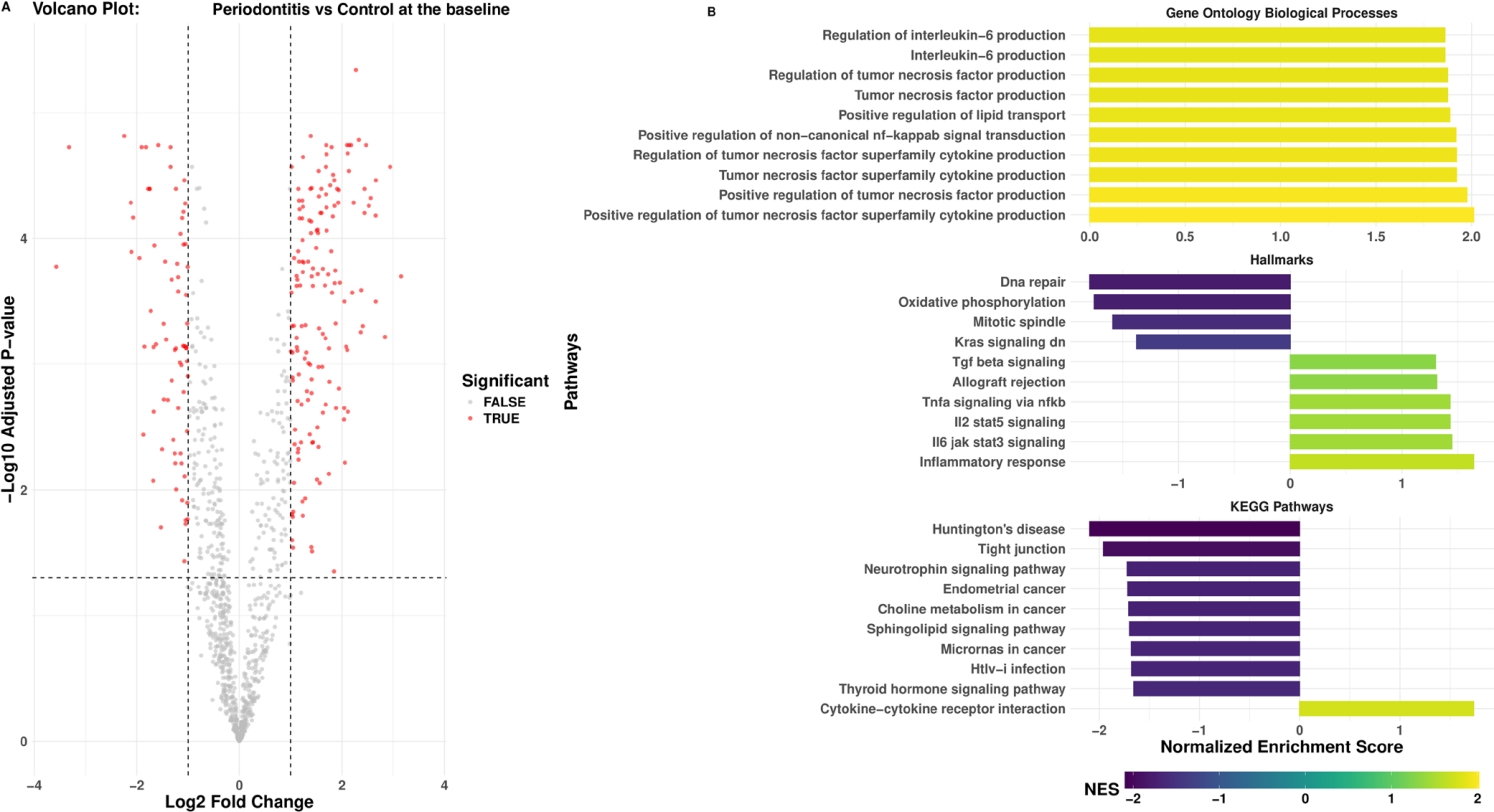
Differential proteins expression and functional analysis in periodontitis vs. control samples. (A) Volcano plot illustrates the differentially expressed proteins (|Log2FC| > 1, adjusted *p*‐value < 0.05) in red color. Horizontal line refers to statistical threshold, while vertical lines to Log2FC thresholds (−1, 1). (B) Top 10 pathways based on Normalized Enrichment Score (NES) for the three categories. Positive NES is associated with positive enrichment in periodontitis samples, while negative NES is connected to positive enrichment in control samples.

To identify a robust candidate signature, Elastic Net classification model was applied. The multivariate model achieved an Area Under the Curve (AUC) of 0.934. Following stringent prioritization (proteins>LOD in 90% of samples and Log2FC>Control IQR), we distilled a final panel of 34 proteins. This panel discriminated furcation defects from health with a sensitivity of 82.9% and specificity of 86.1% (AUC = 0.887).

While the top individual (univariate) performers included CD63 (AUC 0.842), GLIPR1 (AUC 0.792), and IRAG2 (AUC 0.790) (Table 1), the multivariate feature importance analysis of the final 34‐protein model (Table S2 and Figure S1) highlighted GLIPR1, IRAG2, MME (Neprilysin), and IL1B as the strongest collective drivers of the furcation‐associated signature. The Principal Component Analysis (PCA) results are shown in Figure S2.

**TABLE 1:** Top 10 biomarkers with the best single‐protein performance in periodontitis versus control models.

Biomarkers	TP	TN	FP	FN	AUC	Accuracy	Accuracy 95% CI	Sensitivity	Sensitivity 95% CI	Specificity	Specificity 95% CI
CD63	27	29	7	8	0.842	0.789	0.676–0.877	0.771	0.599–0.896	0.806	0.64–0.918
GLIPR1	25	29	7	10	0.792	0.761	0.645–0.854	0.714	0.537–0.854	0.806	0.64–0.918
ACAA1	26	27	9	9	0.791	0.746	0.629–0.842	0.743	0.567–0.875	0.75	0.578–0.879
IRAG2	23	31	5	12	0.79	0.761	0.645–0.854	0.657	0.478–0.809	0.861	0.705–0.953
WIF1	29	25	11	6	0.777	0.761	0.645–0.854	0.829	0.664–0.934	0.694	0.519–0.837
SPRR3	20	32	4	15	0.775	0.732	0.614–0.831	0.571	0.394–0.737	0.889	0.739–0.969
EIF2S2	25	27	9	10	0.769	0.732	0.614–0.831	0.714	0.537–0.854	0.75	0.578–0.879
FCAR	31	22	14	4	0.768	0.746	0.629–0.842	0.886	0.733–0.968	0.611	0.435–0.769
PSME1	21	34	2	14	0.762	0.775	0.66–0.865	0.6	0.421–0.761	0.944	0.813–0.993
ADAM8	28	25	11	7	0.741	0.746	0.629–0.842	0.8	0.631–0.916	0.694	0.519–0.837

## 4 Discussion

This study provides the first high‐multiplex targeted proteomic characterization of Grade II furcation defects. The identified signature reflects a state of intense, unresolved inflammation [6]. The top contributors to our model, such as MME (Neprilysin) and GLIPR1, are biologically consistent with a neutrophil‐dominated lesion. MME is a key neutral endopeptidase expressed by neutrophils, while GLIPR1 is involved in immune regulation and apoptosis. These findings suggest the ‘furcation signature’ is driven by the ineffective host defense typical of deep pockets, resulting in a cycle of molecular dysregulation and persistent inflammation [7, 8].

Furthermore, the prominence of CD63—the strongest single‐protein predictor in our univariate analysis—highlights the intense activation of innate immune cells and exosome‐mediated signaling within the furcation microenvironment. Elevated levels of bone‐resorption mediators (e.g., IL18R1, IL1B) were also observed, confirming the active osteolytic nature of these defects. The pathways enriched in the present analysis—specifically cytokine‐receptor interaction and NF‐kappa B signaling—align with previous proteomic studies showing a shift in inflammatory mediators during the transition from health to periodontitis [6]. However, unlike studies focusing on short‐term active progression [4], these findings likely represent the chronic, established molecular footprint of anatomical defects.

This study is exploratory. The sample size (*N* = 35 pairs), while sufficient for discovery, requires validation in larger, independent cohorts. A primary limitation is the intra‐patient control design. Because shallow posterior sites were used as controls, the identified signature likely reflects severe periodontal inflammation rather than a process unique solely to the furcation anatomy [9]. Future studies must compare furcation defects to non‐furcation intrabony defects and account for clinical variables such as horizontal/vertical bone loss and root trunk anatomy to definitively isolate anatomy‐specific markers. While the absence of a completely healthy (non‐periodontitis) patient cohort is a limitation of this exploratory study, the utilization of an intra‐patient, paired‐site design serves as a significant methodological strength. This split‐mouth approach effectively controls for inter‐individual systemic, genetic, and environmental confounding factors, thereby allowing for the precise isolation of local, site‐level proteomic differences driven by the defect's severe inflammatory burden.

In conclusion, GCF from Grade II furcation defects exhibits a distinct proteomic profile. The identified 34‐protein panel provides a foundation for future research into objective risk assessment and monitoring of these challenging clinical lesions.

## Disclosure

*AI statement*: This manuscript did not use artificial intelligence in any capacity.

*Clinical trial registration*: NCT05541614 (clinicaltrials.gov).

## Ethics Statement

The study was approved by the Ethics Committee of the School of Dentistry at the Aristotle University of Thessaloniki, Greece (Protocol No. 155/14‐03‐2022).

## Supporting information


**Appendix S1:** Comprehensive Study Design, Olink Proteomics Methodology, and Patient Demographics.

**Table S1:** Demographic and Clinical Characteristics of the Study Cohort (*N* = 35).

**Table S2:** Protein biomarkers in Gingival Crevicular Fluid (GCF)—Total candidate biomarkers.

**Figure S1:** Biomarkers feature importance from the final machine learning model in periodontitis versus control prediction.

**Figure S2:** Visualization of Principal Component Analysis (PCA) results. Samples are depicted in different colors and shapes based on sample type.

**Supporting information:** jre70108‐sup‐0006‐Supinfo.docx.
