# Supplementary material for: Proteomic Characterization of Grade II Furcation Defects: A Discovery‐Phase Study
Source: J Periodontal Res. 2026 Apr 9;61(8):978–80. doi: 10.1111/jre.70108 (PMC13703785; doi:10.1111/jre.70108)
Supplement: Supplementary file 1 [file JRE-61-978-s002.docx]

Supplementary Table S2: Protein biomarkers in Gingival Crevicular Fluid (GCF) - Total candidate biomarkers

|  | **Univariate Analysis** | | | | | **ROC Analysis** | | | | | | | | | | | | **Multivariate Modelling** | | | **Biomarkers Prioritization: LOD** | | | **Biomarkers Prioritization: Interquartile Range** | |
| --- | --- | --- | --- | --- | --- | --- | --- | --- | --- | --- | --- | --- | --- | --- | --- | --- | --- | --- | --- | --- | --- | --- | --- | --- | --- |
| **Biomarkers** | **Log2 Fold Change (log2FC)** | **t-statistic** | **p-value** | **Adjusted p-value** | **Univariate Candidate** | **TP** | **TN** | **FP** | **FN** | **AUC** | **Accuracy** | **Accuracy 95% CI** | **Sensitivity** | **Sensitivity 95% CI** | **Specificity** | **Specificity 95% CI** | **ROC candidate** | **Feature Importance** | **Number of machine learning models selected this biomarker as important variable (Important > 2)** | **Multivariate Candidate** | **LOD** | **Quartile of 90% of Diseased samples** | **Expression of 90% of Diseased Samples over the LOD** | **IQR of Healthy samples** | **Diseased vs Healthy Log2FC over IQR of Healthy subjects** |
| CSF2RB | 1.388413127 | 7.07989697 | 7.9891E-10 | 3.35775E-07 | TRUE | 31 | 9 | 27 | 4 | 0.544 | 0.563 | 0.44-0.681 | 0.886 | 0.733-0.968 | 0.25 | 0.121-0.422 | FALSE | 0.014919156 | 1 | FALSE | -0.74810295 | -0.121354006 | 1 | 0.913105169 | TRUE |
| IGF1R | 2.133204543 | 7.04125671 | 9.413E-10 | 3.35775E-07 | TRUE | 14 | 33 | 3 | 21 | 0.655 | 0.662 | 0.54-0.77 | 0.4 | 0.239-0.579 | 0.917 | 0.775-0.982 | FALSE | 0.005666958 | 5 | TRUE | -3.60509927 | 1.116303797 | 1 | 2.044061465 | TRUE |
| FCAR | 2.176511888 | 7.00226494 | 1.1106E-09 | 3.35775E-07 | TRUE | 31 | 22 | 14 | 4 | 0.768 | 0.746 | 0.629-0.842 | 0.886 | 0.733-0.968 | 0.611 | 0.435-0.769 | TRUE | 0.03177772 | 5 | TRUE | -3.56064126 | 4.151344227 | 1 | 2.360949257 | FALSE |
| CD63 | 2.337804406 | 6.96507102 | 1.3002E-09 | 3.35775E-07 | TRUE | 27 | 29 | 7 | 8 | 0.842 | 0.789 | 0.676-0.877 | 0.771 | 0.599-0.896 | 0.806 | 0.64-0.918 | TRUE | 0.069962713 | 5 | TRUE | -4.18280575 | -2.926075679 | 1 | 1.930342046 | TRUE |
| CSF3R | 1.709256446 | 6.85069702 | 2.1093E-09 | 4.35787E-07 | TRUE | 16 | 23 | 13 | 19 | 0.483 | 0.549 | 0.427-0.668 | 0.457 | 0.288-0.634 | 0.639 | 0.462-0.792 | FALSE |  |  |  | -1.21065773 | -0.498174418 | 1 | 1.142563564 | TRUE |
| IRAG2 | 2.262256209 | 6.80720733 | 2.5345E-09 | 4.36356E-07 | TRUE | 23 | 31 | 5 | 12 | 0.79 | 0.761 | 0.645-0.854 | 0.657 | 0.478-0.809 | 0.861 | 0.705-0.953 | TRUE | 0.043239592 | 4 | TRUE | -2.45702926 | 0.063740624 | 1 | 1.661397098 | TRUE |
| SERPINB5 | -3.340510887 | -6.76559885 | 3.0207E-09 | 4.45768E-07 | FALSE | 26 | 26 | 10 | 9 | 0.739 | 0.732 | 0.614-0.831 | 0.743 | 0.567-0.875 | 0.722 | 0.548-0.858 | TRUE |  |  |  | 0.91189567 | -3.457153176 | 0 | 2.817147268 | FALSE |
| CLEC4D | 2.107839917 | 6.60761567 | 5.8706E-09 | 6.74771E-07 | TRUE | 19 | 29 | 7 | 16 | 0.596 | 0.676 | 0.555-0.782 | 0.543 | 0.366-0.712 | 0.806 | 0.64-0.918 | FALSE | 0.012799033 | 1 | FALSE | -0.35804315 | 2.725385411 | 1 | 1.515215773 | TRUE |
| CD244 | 1.236933549 | 6.58928228 | 6.3399E-09 | 6.74771E-07 | TRUE | 1 | 35 | 1 | 34 | 0.318 | 0.507 | 0.386-0.628 | 0.029 | 0.001-0.149 | 0.972 | 0.855-0.999 | FALSE | 0.015392129 | 3 | TRUE | -4.94049151 | -4.958788501 | 0 | 1.289784616 | FALSE |
| IL18R1 | 1.670257559 | 6.56390252 | 7.0516E-09 | 6.74771E-07 | TRUE | 31 | 19 | 17 | 4 | 0.67 | 0.704 | 0.584-0.807 | 0.886 | 0.733-0.968 | 0.528 | 0.355-0.696 | FALSE | 0.023155672 | 4 | TRUE | -5.14670352 | -2.638376079 | 1 | 1.295386544 | TRUE |
| TPR | 2.48001217 | 6.55941742 | 7.1854E-09 | 6.74771E-07 | TRUE | 25 | 26 | 10 | 10 | 0.722 | 0.718 | 0.599-0.819 | 0.714 | 0.537-0.854 | 0.722 | 0.548-0.858 | TRUE | 0.020818833 | 4 | TRUE | -1.23426835 | 3.805049756 | 1 | 1.846018548 | TRUE |
| MAD1L1 | 2.175357591 | 6.502141 | 9.1322E-09 | 7.25655E-07 | TRUE | 27 | 23 | 13 | 8 | 0.706 | 0.704 | 0.584-0.807 | 0.771 | 0.599-0.896 | 0.639 | 0.462-0.792 | TRUE | 0.011932216 | 4 | TRUE | -3.07076502 | 4.079899208 | 1 | 1.788508857 | TRUE |
| CLEC5A | 1.815117525 | 6.37534129 | 1.5501E-08 | 9.93823E-07 | TRUE | 31 | 8 | 28 | 4 | 0.533 | 0.549 | 0.427-0.668 | 0.886 | 0.733-0.968 | 0.222 | 0.101-0.392 | FALSE |  |  |  | -7.55035363 | 3.790405058 | 1 | 1.610035199 | TRUE |
| SIGLEC7 | 1.697441463 | 6.36576621 | 1.6132E-08 | 9.93823E-07 | TRUE | 4 | 36 | 0 | 31 | 0.494 | 0.563 | 0.44-0.681 | 0.114 | 0.032-0.267 | 1 | 0.903-1 | FALSE |  |  |  | -7.03755512 | -3.004465956 | 1 | 1.378117979 | TRUE |
| SEL1L | 1.260630807 | 6.35576962 | 1.6817E-08 | 9.93823E-07 | TRUE | 17 | 29 | 7 | 18 | 0.62 | 0.648 | 0.525-0.758 | 0.486 | 0.314-0.66 | 0.806 | 0.64-0.918 | FALSE | 0.006655649 | 2 | FALSE | -1.17102749 | -0.837847052 | 1 | 1.056467933 | TRUE |
| LY75 | 1.582266299 | 6.34871842 | 1.7317E-08 | 9.93823E-07 | TRUE | 21 | 25 | 11 | 14 | 0.618 | 0.648 | 0.525-0.758 | 0.6 | 0.421-0.761 | 0.694 | 0.519-0.837 | FALSE | 0.007342338 | 3 | TRUE | -2.84965126 | -3.138006242 | 0 | 1.159633091 | TRUE |
| TGFB1 | 1.428811161 | 6.32629203 | 1.901E-08 | 1.03353E-06 | TRUE | 21 | 25 | 11 | 14 | 0.644 | 0.648 | 0.525-0.758 | 0.6 | 0.421-0.761 | 0.694 | 0.519-0.837 | FALSE | 0.003268587 | 1 | FALSE | 0.02809363 | -1.443157663 | 0 | 1.015854033 | TRUE |
| SPRR3 | -1.734590129 | -6.24113095 | 2.7066E-08 | 1.27672E-06 | FALSE | 20 | 32 | 4 | 15 | 0.775 | 0.732 | 0.614-0.831 | 0.571 | 0.394-0.737 | 0.889 | 0.739-0.969 | TRUE |  |  |  | -2.02339848 | -1.807266853 | 1 | 1.271697033 | FALSE |
| CEACAM3 | 1.394793473 | 6.23543338 | 2.7712E-08 | 1.27672E-06 | TRUE | 23 | 19 | 17 | 12 | 0.535 | 0.592 | 0.468-0.707 | 0.657 | 0.478-0.809 | 0.528 | 0.355-0.696 | FALSE | 0.006885609 | 2 | FALSE | -0.63087054 | 3.90120153 | 1 | 1.633535712 | FALSE |
| IFI30 | 1.702954923 | 6.22928821 | 2.8427E-08 | 1.27672E-06 | TRUE | 14 | 26 | 10 | 21 | 0.528 | 0.563 | 0.44-0.681 | 0.4 | 0.239-0.579 | 0.722 | 0.548-0.858 | FALSE | 0.010916852 | 1 | FALSE | -2.80531966 | 2.702988966 | 1 | 1.284243164 | TRUE |
| CEMIP2 | 1.022340341 | 6.18598432 | 3.4003E-08 | 1.46354E-06 | TRUE | 35 | 1 | 35 | 0 | 0.394 | 0.507 | 0.386-0.628 | 1 | 0.9-1 | 0.028 | 0.001-0.145 | FALSE |  |  |  | -2.23786437 | -2.956381689 | 0 | 0.769024737 | TRUE |
| RNF41 | 2.959593819 | 6.08263632 | 5.2071E-08 | 2.06881E-06 | TRUE | 24 | 25 | 11 | 11 | 0.714 | 0.69 | 0.569-0.795 | 0.686 | 0.507-0.831 | 0.694 | 0.519-0.837 | TRUE | 0.020014035 | 4 | TRUE | 2.56240084 | 1.913998041 | 0 | 3.160304372 | FALSE |
| MUC20 | -1.405874789 | -6.05786208 | 5.7655E-08 | 2.20583E-06 | FALSE | 27 | 24 | 12 | 8 | 0.721 | 0.718 | 0.599-0.819 | 0.771 | 0.599-0.896 | 0.667 | 0.49-0.814 | TRUE |  |  |  | -3.89057695 | -8.268186513 | 0 | 1.170881014 | FALSE |
| CALCOCO2 | 1.817206196 | 5.98891593 | 7.6505E-08 | 2.60498E-06 | TRUE | 25 | 23 | 13 | 10 | 0.687 | 0.676 | 0.555-0.782 | 0.714 | 0.537-0.854 | 0.639 | 0.462-0.792 | FALSE | 0.022520391 | 4 | TRUE | -1.41868621 | 2.154292845 | 1 | 1.387405879 | TRUE |
| DNAJA4 | 1.867429659 | 5.98886653 | 7.6521E-08 | 2.60498E-06 | TRUE | 23 | 23 | 13 | 12 | 0.657 | 0.648 | 0.525-0.758 | 0.657 | 0.478-0.809 | 0.639 | 0.462-0.792 | FALSE | 0.015164189 | 4 | TRUE | -3.31029807 | 0.936438432 | 1 | 1.904781893 | FALSE |
| PTP4A3 | 1.185996304 | 5.98534814 | 7.7632E-08 | 2.60498E-06 | TRUE | 18 | 29 | 7 | 17 | 0.637 | 0.662 | 0.54-0.77 | 0.514 | 0.34-0.686 | 0.806 | 0.64-0.918 | FALSE | 0.012678007 | 4 | TRUE | -1.25744657 | -1.86698935 | 0 | 0.972089923 | TRUE |
| OSM | 2.112896527 | 5.98364583 | 7.8175E-08 | 2.60498E-06 | TRUE | 13 | 33 | 3 | 22 | 0.631 | 0.648 | 0.525-0.758 | 0.371 | 0.215-0.551 | 0.917 | 0.775-0.982 | FALSE | 0.010130436 | 1 | FALSE | -3.51837352 | 4.329416891 | 1 | 2.159290457 | FALSE |
| ADAM8 | 1.554031562 | 5.87647749 | 1.2111E-07 | 3.57445E-06 | TRUE | 28 | 25 | 11 | 7 | 0.741 | 0.746 | 0.629-0.842 | 0.8 | 0.631-0.916 | 0.694 | 0.519-0.837 | TRUE | 0.033693063 | 4 | TRUE | -3.77316277 | 2.295559837 | 1 | 1.559501683 | FALSE |
| MME | 2.412324173 | 5.81774842 | 1.5379E-07 | 4.30433E-06 | TRUE | 25 | 26 | 10 | 10 | 0.741 | 0.718 | 0.599-0.819 | 0.714 | 0.537-0.854 | 0.722 | 0.548-0.858 | TRUE | 0.022886455 | 5 | TRUE | -4.39408663 | -3.158400153 | 1 | 2.044057372 | TRUE |
| DPEP2 | 2.454626469 | 5.80458457 | 1.6223E-07 | 4.41011E-06 | TRUE | 30 | 16 | 20 | 5 | 0.67 | 0.648 | 0.525-0.758 | 0.857 | 0.697-0.952 | 0.444 | 0.279-0.619 | FALSE | 0.008474867 | 3 | TRUE | -3.86920458 | -4.133347505 | 0 | 1.994340712 | TRUE |
| ERP44 | 1.581401045 | 5.78551802 | 1.7528E-07 | 4.52649E-06 | TRUE | 25 | 16 | 20 | 10 | 0.549 | 0.577 | 0.454-0.694 | 0.714 | 0.537-0.854 | 0.444 | 0.279-0.619 | FALSE | 0.010095958 | 2 | FALSE | -3.02779712 | -0.133492058 | 1 | 1.962318389 | FALSE |
| GLIPR1 | 1.704844721 | 5.77525757 | 1.8272E-07 | 4.60362E-06 | TRUE | 25 | 29 | 7 | 10 | 0.792 | 0.761 | 0.645-0.854 | 0.714 | 0.537-0.854 | 0.806 | 0.64-0.918 | TRUE | 0.040434472 | 4 | TRUE | -5.04887016 | -4.549468707 | 1 | 1.21570035 | TRUE |
| EML4 | 2.596519424 | 5.76558886 | 1.9002E-07 | 4.67352E-06 | TRUE | 29 | 17 | 19 | 6 | 0.651 | 0.648 | 0.525-0.758 | 0.829 | 0.664-0.934 | 0.472 | 0.304-0.645 | FALSE |  |  |  | -4.29113763 | -3.260524353 | 1 | 2.019841301 | TRUE |
| HGF | 1.91927551 | 5.74797341 | 2.0406E-07 | 4.87872E-06 | TRUE | 29 | 16 | 20 | 6 | 0.602 | 0.634 | 0.511-0.745 | 0.829 | 0.664-0.934 | 0.444 | 0.279-0.619 | FALSE | 0.010543149 | 3 | TRUE | -4.50761886 | -1.260681402 | 1 | 2.178348761 | FALSE |
| SIGLEC10 | 1.246556815 | 5.7061064 | 2.4168E-07 | 5.54791E-06 | TRUE | 34 | 2 | 34 | 1 | 0.312 | 0.507 | 0.386-0.628 | 0.971 | 0.851-0.999 | 0.056 | 0.007-0.187 | FALSE |  |  |  | -2.32465891 | -1.446721354 | 1 | 1.135021039 | TRUE |
| CSF1 | 1.229319609 | 5.67384724 | 2.7526E-07 | 6.08017E-06 | TRUE | 29 | 22 | 14 | 6 | 0.657 | 0.718 | 0.599-0.819 | 0.829 | 0.664-0.934 | 0.611 | 0.435-0.769 | FALSE | 0.011343879 | 4 | TRUE | -7.62926412 | -4.036801012 | 1 | 1.368027373 | FALSE |
| WIF1 | 1.935942035 | 5.66737934 | 2.8252E-07 | 6.08017E-06 | TRUE | 29 | 25 | 11 | 6 | 0.777 | 0.761 | 0.645-0.854 | 0.829 | 0.664-0.934 | 0.694 | 0.519-0.837 | TRUE | 0.027305845 | 5 | TRUE | -8.37694798 | -4.983128957 | 1 | 1.967554377 | FALSE |
| STX8 | 1.173538422 | 5.65853378 | 2.9277E-07 | 6.17205E-06 | TRUE | 15 | 27 | 9 | 20 | 0.552 | 0.592 | 0.468-0.707 | 0.429 | 0.263-0.606 | 0.75 | 0.578-0.879 | FALSE | 0.017079255 | 4 | TRUE | -0.26050136 | -0.077601046 | 1 | 1.100699874 | TRUE |
| BSG | 1.52339819 | 5.63628255 | 3.2018E-07 | 6.61502E-06 | TRUE | 25 | 28 | 8 | 10 | 0.701 | 0.746 | 0.629-0.842 | 0.714 | 0.537-0.854 | 0.778 | 0.608-0.899 | TRUE | 0.00324315 | 3 | TRUE | -4.88733814 | -3.504619361 | 1 | 1.60628208 | FALSE |
| TNFSF14 | 2.616954903 | 5.61652776 | 3.4663E-07 | 7.02105E-06 | TRUE | 18 | 27 | 9 | 17 | 0.591 | 0.634 | 0.511-0.745 | 0.514 | 0.34-0.686 | 0.75 | 0.578-0.879 | FALSE |  |  |  | -5.23870311 | -0.152707834 | 1 | 3.496060034 | FALSE |
| ZER1 | 2.691995371 | 5.58419816 | 3.9464E-07 | 7.6917E-06 | TRUE | 17 | 30 | 6 | 18 | 0.631 | 0.662 | 0.54-0.77 | 0.486 | 0.314-0.66 | 0.833 | 0.672-0.936 | FALSE | 0.011733728 | 2 | FALSE | -0.19756822 | 0.207890535 | 1 | 2.223143718 | TRUE |
| CLEC7A | 1.412538179 | 5.57497347 | 4.095E-07 | 7.8335E-06 | TRUE | 1 | 35 | 1 | 34 | 0.292 | 0.507 | 0.386-0.628 | 0.029 | 0.001-0.149 | 0.972 | 0.855-0.999 | FALSE |  |  |  | -2.0592362 | -0.776619394 | 1 | 1.152424136 | TRUE |
| TNFSF12 | 1.896510823 | 5.51808261 | 5.1409E-07 | 9.0064E-06 | TRUE | 18 | 29 | 7 | 17 | 0.661 | 0.662 | 0.54-0.77 | 0.514 | 0.34-0.686 | 0.806 | 0.64-0.918 | FALSE | 0.014621315 | 4 | TRUE | -4.71705957 | -4.153556314 | 1 | 2.359291243 | FALSE |
| TLR1 | 1.591635749 | 5.50216412 | 5.478E-07 | 9.27663E-06 | TRUE | 29 | 19 | 17 | 6 | 0.697 | 0.676 | 0.555-0.782 | 0.829 | 0.664-0.934 | 0.528 | 0.355-0.696 | FALSE | 0.018147566 | 4 | TRUE | -1.08652324 | -0.521179737 | 1 | 1.361314426 | TRUE |
| CCAR1 | 2.253426271 | 5.48380745 | 5.8937E-07 | 9.7116E-06 | TRUE | 22 | 26 | 10 | 13 | 0.664 | 0.676 | 0.555-0.782 | 0.629 | 0.449-0.785 | 0.722 | 0.548-0.858 | FALSE | 0.007481366 | 2 | FALSE | -2.42536342 | 3.532741173 | 1 | 2.079716717 | TRUE |
| PSTPIP1 | 1.843791042 | 5.48256895 | 5.9229E-07 | 9.7116E-06 | TRUE | 27 | 24 | 12 | 8 | 0.661 | 0.718 | 0.599-0.819 | 0.771 | 0.599-0.896 | 0.667 | 0.49-0.814 | FALSE | 0.020669485 | 4 | TRUE | -6.16460817 | -1.267740527 | 1 | 2.282757854 | FALSE |
| FMNL1 | 1.848404813 | 5.46087926 | 6.4568E-07 | 1.03208E-05 | TRUE | 32 | 14 | 22 | 3 | 0.524 | 0.648 | 0.525-0.758 | 0.914 | 0.769-0.982 | 0.389 | 0.231-0.565 | FALSE | 0.009268475 | 2 | FALSE | -2.57871504 | -0.261584726 | 1 | 2.252868176 | FALSE |
| DNMBP | 1.727013021 | 5.45942618 | 6.4942E-07 | 1.03208E-05 | TRUE | 7 | 31 | 5 | 28 | 0.473 | 0.535 | 0.413-0.655 | 0.2 | 0.084-0.369 | 0.861 | 0.705-0.953 | FALSE |  |  |  | -3.22541201 | 2.933703606 | 1 | 1.904331364 | FALSE |
| FBXO28 | 1.154920279 | 5.44825725 | 6.789E-07 | 1.06258E-05 | TRUE | 35 | 3 | 33 | 0 | 0.487 | 0.535 | 0.413-0.655 | 1 | 0.9-1 | 0.083 | 0.018-0.225 | FALSE |  |  |  | 0.29078914 | 0.60878902 | 1 | 0.789285993 | TRUE |
| ENPP1 | 1.077848125 | 5.40120198 | 8.1824E-07 | 1.243E-05 | TRUE | 3 | 35 | 1 | 32 | 0.359 | 0.535 | 0.413-0.655 | 0.086 | 0.018-0.231 | 0.972 | 0.855-0.999 | FALSE |  |  |  | -6.45037111 | -7.163672787 | 0 | 1.046513353 | TRUE |
| IL1B | 1.716056858 | 5.39390306 | 8.4224E-07 | 1.26091E-05 | TRUE | 28 | 20 | 16 | 7 | 0.683 | 0.676 | 0.555-0.782 | 0.8 | 0.631-0.916 | 0.556 | 0.381-0.721 | FALSE | 0.01380481 | 5 | TRUE | -1.47155863 | 7.512977069 | 1 | 1.673331295 | TRUE |
| CLEC12A | 1.798989657 | 5.3525573 | 9.918E-07 | 1.443E-05 | TRUE | 22 | 27 | 9 | 13 | 0.711 | 0.69 | 0.569-0.795 | 0.629 | 0.449-0.785 | 0.75 | 0.578-0.879 | TRUE | 0.014643356 | 4 | TRUE | -2.24303322 | 0.508482556 | 1 | 2.221434404 | FALSE |
| IDUA | 1.328106128 | 5.33885289 | 1.0469E-06 | 1.50202E-05 | TRUE | 28 | 15 | 21 | 7 | 0.603 | 0.606 | 0.483-0.72 | 0.8 | 0.631-0.916 | 0.417 | 0.255-0.592 | FALSE | 0.010661109 | 4 | TRUE | -4.5787945 | -0.382615076 | 1 | 1.532648031 | FALSE |
| VEGFA | 1.151936144 | 5.33163769 | 1.0771E-06 | 1.5242E-05 | TRUE | 14 | 29 | 7 | 21 | 0.602 | 0.606 | 0.483-0.72 | 0.4 | 0.239-0.579 | 0.806 | 0.64-0.918 | FALSE | 0.001863304 | 1 | FALSE | -6.6613039 | 1.238559918 | 1 | 1.068675798 | TRUE |
| TNFRSF10B | 1.266209518 | 5.30519229 | 1.1954E-06 | 1.66866E-05 | TRUE | 31 | 7 | 29 | 4 | 0.441 | 0.535 | 0.413-0.655 | 0.886 | 0.733-0.968 | 0.194 | 0.082-0.36 | FALSE |  |  |  | -5.85945671 | -1.687824755 | 1 | 1.100372411 | TRUE |
| VWA1 | 1.135887437 | 5.28869339 | 1.2755E-06 | 1.75677E-05 | TRUE | 26 | 21 | 15 | 9 | 0.625 | 0.662 | 0.54-0.77 | 0.743 | 0.567-0.875 | 0.583 | 0.408-0.745 | FALSE | 0.017417985 | 3 | TRUE | -4.21540856 | -1.679668158 | 1 | 1.136755957 | FALSE |
| VAV3 | 1.386293314 | 5.27677556 | 1.3366E-06 | 1.79043E-05 | TRUE | 30 | 15 | 21 | 5 | 0.633 | 0.634 | 0.511-0.745 | 0.857 | 0.697-0.952 | 0.417 | 0.255-0.592 | FALSE |  |  |  | 1.09481945 | 1.519747417 | 1 | 1.456731843 | FALSE |
| RNF149 | 1.145503286 | 5.27557052 | 1.343E-06 | 1.79043E-05 | TRUE | 35 | 3 | 33 | 0 | 0.333 | 0.535 | 0.413-0.655 | 1 | 0.9-1 | 0.083 | 0.018-0.225 | FALSE |  |  |  | 0.00569041 | -0.482402166 | 0 | 1.239240223 | FALSE |
| UBL4A | 2.570340043 | 5.27387765 | 1.3519E-06 | 1.79043E-05 | TRUE | 33 | 9 | 27 | 2 | 0.598 | 0.592 | 0.468-0.707 | 0.943 | 0.808-0.993 | 0.25 | 0.121-0.422 | FALSE |  |  |  | -3.16535182 | -1.720049893 | 1 | 2.930999824 | FALSE |
| SLMAP | 1.538368614 | 5.22376061 | 1.6453E-06 | 2.07268E-05 | TRUE | 34 | 3 | 33 | 1 | 0.414 | 0.521 | 0.399-0.641 | 0.971 | 0.851-0.999 | 0.083 | 0.018-0.225 | FALSE |  |  |  | -4.26293145 | -0.903122494 | 1 | 1.493678377 | TRUE |
| SIGLEC9 | 1.599903818 | 5.20411522 | 1.7766E-06 | 2.21114E-05 | TRUE | 11 | 31 | 5 | 24 | 0.567 | 0.592 | 0.468-0.707 | 0.314 | 0.169-0.493 | 0.861 | 0.705-0.953 | FALSE |  |  |  | -4.20229169 | -2.149990272 | 1 | 1.711184795 | FALSE |
| UBAC1 | 1.265718916 | 5.17446785 | 1.9945E-06 | 2.42388E-05 | TRUE | 17 | 23 | 13 | 18 | 0.499 | 0.563 | 0.44-0.681 | 0.486 | 0.314-0.66 | 0.639 | 0.462-0.792 | FALSE |  |  |  | -2.42449483 | -1.467844497 | 1 | 1.553358482 | FALSE |
| PRRC1 | 1.454631381 | 5.16220203 | 2.0921E-06 | 2.51294E-05 | TRUE | 2 | 34 | 2 | 33 | 0.267 | 0.507 | 0.386-0.628 | 0.057 | 0.007-0.192 | 0.944 | 0.813-0.993 | FALSE |  |  |  | -1.34184 | -2.261852381 | 0 | 1.41034315 | TRUE |
| CRACR2A | 1.802302841 | 5.1060095 | 2.6025E-06 | 2.95426E-05 | TRUE | 34 | 5 | 31 | 1 | 0.54 | 0.549 | 0.427-0.668 | 0.971 | 0.851-0.999 | 0.139 | 0.047-0.295 | FALSE | 0.001117457 | 3 | TRUE | -2.19869073 | 2.979659598 | 1 | 1.898787667 | FALSE |
| MORC3 | 1.263168055 | 5.09136841 | 2.7544E-06 | 3.06624E-05 | TRUE | 28 | 15 | 21 | 7 | 0.544 | 0.606 | 0.483-0.72 | 0.8 | 0.631-0.916 | 0.417 | 0.255-0.592 | FALSE | 0.015055509 | 1 | FALSE | 0.1750822 | 2.96493679 | 1 | 1.66222101 | FALSE |
| TCERG1 | 1.656397729 | 5.05260897 | 3.1996E-06 | 3.51619E-05 | TRUE | 22 | 28 | 8 | 13 | 0.689 | 0.704 | 0.584-0.807 | 0.629 | 0.449-0.785 | 0.778 | 0.608-0.899 | FALSE | 0.001129227 | 1 | FALSE | -3.63874853 | 0.435818649 | 1 | 1.838770539 | FALSE |
| ADGRE1 | 1.098386903 | 5.0158381 | 3.6868E-06 | 4.0089E-05 | TRUE | 35 | 0 | 36 | 0 | 0.331 | 0.493 | 0.372-0.614 | 1 | 0.9-1 | 0 | 0-0.097 | FALSE |  |  |  | -2.30810216 | -2.402249834 | 0 | 1.300435536 | FALSE |
| CNP | 2.240927279 | 4.99586329 | 3.9811E-06 | 4.23017E-05 | TRUE | 29 | 19 | 17 | 6 | 0.693 | 0.676 | 0.555-0.782 | 0.829 | 0.664-0.934 | 0.528 | 0.355-0.696 | FALSE | 0.00916016 | 5 | TRUE | -2.51938749 | 0.894481145 | 1 | 2.873560969 | FALSE |
| ADAM15 | 1.148372418 | 4.98434241 | 4.1612E-06 | 4.34194E-05 | TRUE | 11 | 30 | 6 | 24 | 0.542 | 0.577 | 0.454-0.694 | 0.314 | 0.169-0.493 | 0.833 | 0.672-0.936 | FALSE | 0.007002393 | 2 | FALSE | -2.14547551 | -1.802124221 | 1 | 1.160634058 | FALSE |
| AP3B1 | 1.874308452 | 4.97291927 | 4.3476E-06 | 4.49108E-05 | TRUE | 24 | 26 | 10 | 11 | 0.697 | 0.704 | 0.584-0.807 | 0.686 | 0.507-0.831 | 0.722 | 0.548-0.858 | FALSE | 0.015027779 | 3 | TRUE | -2.15549764 | -0.696617715 | 1 | 2.522533237 | FALSE |
| LRRC25 | 1.515309828 | 4.96380165 | 4.5022E-06 | 4.60476E-05 | TRUE | 4 | 35 | 1 | 31 | 0.499 | 0.549 | 0.427-0.668 | 0.114 | 0.032-0.267 | 0.972 | 0.855-0.999 | FALSE |  |  |  | -3.21881995 | -0.492577837 | 1 | 1.415585088 | TRUE |
| NPM1 | 1.309582375 | 4.95984663 | 4.571E-06 | 4.62923E-05 | TRUE | 35 | 1 | 35 | 0 | 0.342 | 0.507 | 0.386-0.628 | 1 | 0.9-1 | 0.028 | 0.001-0.145 | FALSE |  |  |  | 0.54884356 | 0.538916989 | 0 | 0.751516257 | TRUE |
| PSIP1 | 1.391327052 | 4.94100208 | 4.9128E-06 | 4.92713E-05 | TRUE | 16 | 26 | 10 | 19 | 0.539 | 0.592 | 0.468-0.707 | 0.457 | 0.288-0.634 | 0.722 | 0.548-0.858 | FALSE |  |  |  | -1.31137518 | -1.790191574 | 0 | 1.453022916 | FALSE |
| TAX1BP1 | 1.44861346 | 4.93036546 | 5.1167E-06 | 5.08223E-05 | TRUE | 1 | 36 | 0 | 34 | 0.333 | 0.521 | 0.399-0.641 | 0.029 | 0.001-0.149 | 1 | 0.903-1 | FALSE |  |  |  | -1.47695004 | 1.159393735 | 1 | 1.392639763 | TRUE |
| ZC3H12A | 2.412975629 | 4.91840231 | 5.3558E-06 | 5.26912E-05 | TRUE | 13 | 29 | 7 | 22 | 0.555 | 0.592 | 0.468-0.707 | 0.371 | 0.215-0.551 | 0.806 | 0.64-0.918 | FALSE |  |  |  | -0.19401929 | -0.336639228 | 0 | 2.745908703 | FALSE |
| PRKAR2A | 1.866445388 | 4.89787225 | 5.792E-06 | 5.63142E-05 | TRUE | 22 | 23 | 13 | 13 | 0.567 | 0.634 | 0.511-0.745 | 0.629 | 0.449-0.785 | 0.639 | 0.462-0.792 | FALSE |  |  |  | -2.11170456 | -0.555160967 | 1 | 2.553948166 | FALSE |
| TIMP2 | 2.838361853 | 4.89112208 | 5.9428E-06 | 5.68419E-05 | TRUE | 28 | 23 | 13 | 7 | 0.715 | 0.718 | 0.599-0.819 | 0.8 | 0.631-0.916 | 0.639 | 0.462-0.792 | TRUE | 0.014720026 | 4 | TRUE | -2.57266976 | 1.508813787 | 1 | 3.033193353 | FALSE |
| SUMO1 | -2.093971277 | -4.77998935 | 9.0541E-06 | 8.20429E-05 | FALSE | 28 | 22 | 14 | 7 | 0.717 | 0.704 | 0.584-0.807 | 0.8 | 0.631-0.916 | 0.611 | 0.435-0.769 | TRUE |  |  |  | -1.20263649 | -7.001385106 | 0 | 2.389238816 | FALSE |
| IFNGR2 | 1.210760015 | 4.76643541 | 9.5284E-06 | 8.51396E-05 | TRUE | 22 | 28 | 8 | 13 | 0.7 | 0.704 | 0.584-0.807 | 0.629 | 0.449-0.785 | 0.778 | 0.608-0.899 | FALSE | 0.010312049 | 4 | TRUE | -5.53555314 | -3.77741157 | 1 | 1.202750153 | TRUE |
| IL17A | 1.585071435 | 4.76325596 | 9.6431E-06 | 8.51396E-05 | TRUE | 33 | 11 | 25 | 2 | 0.617 | 0.62 | 0.497-0.732 | 0.943 | 0.808-0.993 | 0.306 | 0.163-0.481 | FALSE | 0.019072206 | 4 | TRUE | -0.91418598 | -1.537655663 | 0 | 1.347414307 | TRUE |
| NFU1 | 2.058571681 | 4.70267123 | 1.2105E-05 | 0.000105968 | TRUE | 19 | 26 | 10 | 16 | 0.613 | 0.634 | 0.511-0.745 | 0.543 | 0.366-0.712 | 0.722 | 0.548-0.858 | FALSE | 0.005459608 | 2 | FALSE | -2.61430432 | 0.028170913 | 1 | 3.264545704 | FALSE |
| SMARCA4 | 1.832246574 | 4.68303476 | 1.3027E-05 | 0.000112767 | TRUE | 16 | 26 | 10 | 19 | 0.537 | 0.592 | 0.468-0.707 | 0.457 | 0.288-0.634 | 0.722 | 0.548-0.858 | FALSE |  |  |  | 0.23407857 | 2.640944083 | 1 | 1.708490722 | TRUE |
| NAGK | 2.370409025 | 4.66921217 | 1.3716E-05 | 0.000116139 | TRUE | 15 | 30 | 6 | 20 | 0.567 | 0.634 | 0.511-0.745 | 0.429 | 0.263-0.606 | 0.833 | 0.672-0.936 | FALSE | 0.005951317 | 1 | FALSE | -3.07925598 | -4.84625192 | 0 | 1.459134424 | TRUE |
| CALML4 | 1.6894219 | 4.65838985 | 1.4281E-05 | 0.000119937 | TRUE | 34 | 2 | 34 | 1 | 0.314 | 0.507 | 0.386-0.628 | 0.971 | 0.851-0.999 | 0.056 | 0.007-0.187 | FALSE |  |  |  | -4.47006577 | -2.915123122 | 1 | 1.859065885 | FALSE |
| GFER | 1.584259235 | 4.61960579 | 1.6496E-05 | 0.000135288 | TRUE | 21 | 26 | 10 | 14 | 0.617 | 0.662 | 0.54-0.77 | 0.6 | 0.421-0.761 | 0.722 | 0.548-0.858 | FALSE | 0.013309055 | 1 | FALSE | -0.93984708 | 2.56513226 | 1 | 1.953275438 | FALSE |
| IKBKG | 1.215683642 | 4.61951881 | 1.6502E-05 | 0.000135288 | TRUE | 31 | 5 | 31 | 4 | 0.352 | 0.507 | 0.386-0.628 | 0.886 | 0.733-0.968 | 0.139 | 0.047-0.295 | FALSE |  |  |  | -1.7913827 | -0.15454415 | 1 | 1.464974253 | FALSE |
| DCTN4 | -1.021276591 | -4.58696934 | 1.8617E-05 | 0.000150243 | FALSE | 34 | 4 | 32 | 1 | 0.43 | 0.535 | 0.413-0.655 | 0.971 | 0.851-0.999 | 0.111 | 0.031-0.261 | FALSE | 0.007504485 | 3 | TRUE | -0.2636123 | -2.521631075 | 0 | 1.14991942 | FALSE |
| FHIT | 1.561057376 | 4.58476023 | 1.877E-05 | 0.000150302 | TRUE | 32 | 8 | 28 | 3 | 0.529 | 0.563 | 0.44-0.681 | 0.914 | 0.769-0.982 | 0.222 | 0.101-0.392 | FALSE |  |  |  | -0.51107963 | -1.131087751 | 0 | 1.26272749 | TRUE |
| APIP | 2.678195582 | 4.57337512 | 1.9576E-05 | 0.000155555 | TRUE | 10 | 33 | 3 | 25 | 0.565 | 0.606 | 0.483-0.72 | 0.286 | 0.146-0.463 | 0.917 | 0.775-0.982 | FALSE |  |  |  | -4.26459931 | -3.559825615 | 1 | 3.765264144 | FALSE |
| RAB2B | 1.031518111 | 4.56862129 | 1.9923E-05 | 0.000157102 | TRUE | 31 | 10 | 26 | 4 | 0.517 | 0.577 | 0.454-0.694 | 0.886 | 0.733-0.968 | 0.278 | 0.142-0.452 | FALSE |  |  |  | -0.07206962 | 0.224421172 | 1 | 0.97847708 | TRUE |
| RANBP2 | 0.892701184 | 4.53099238 | 2.2886E-05 | 0.000179099 | FALSE | 15 | 27 | 9 | 20 | 0.556 | 0.592 | 0.468-0.707 | 0.429 | 0.263-0.606 | 0.75 | 0.578-0.879 | FALSE | 0.013284483 | 4 | TRUE | 0.9292021 | 0.920739773 | 0 | 0.568639105 | TRUE |
| MAPKAPK2 | 1.147604875 | 4.51108081 | 2.4623E-05 | 0.000191242 | TRUE | 30 | 14 | 22 | 5 | 0.571 | 0.62 | 0.497-0.732 | 0.857 | 0.697-0.952 | 0.389 | 0.231-0.565 | FALSE |  |  |  | -1.30920251 | 0.92987266 | 1 | 1.662177985 | FALSE |
| KYAT1 | 3.073555272 | 4.49880237 | 2.5757E-05 | 0.000195637 | TRUE | 22 | 27 | 9 | 13 | 0.702 | 0.69 | 0.569-0.795 | 0.629 | 0.449-0.785 | 0.75 | 0.578-0.879 | TRUE | 0.016263417 | 4 | TRUE | -6.4007745 | -4.402076131 | 1 | 3.645726333 | FALSE |
| VNN2 | 1.164322083 | 4.46454586 | 2.9195E-05 | 0.00021697 | TRUE | 3 | 35 | 1 | 32 | 0.356 | 0.535 | 0.413-0.655 | 0.086 | 0.018-0.231 | 0.972 | 0.855-0.999 | FALSE |  |  |  | -2.76801042 | -0.0469682 | 1 | 1.655675136 | FALSE |
| DUT | 1.74351437 | 4.40452937 | 3.6322E-05 | 0.000259571 | TRUE | 24 | 27 | 9 | 11 | 0.725 | 0.718 | 0.599-0.819 | 0.686 | 0.507-0.831 | 0.75 | 0.578-0.879 | TRUE | 0.014251408 | 4 | TRUE | -0.41187693 | 5.946510712 | 1 | 2.470223869 | FALSE |
| CRYZL1 | 2.093722105 | 4.39500496 | 3.7599E-05 | 0.000266022 | TRUE | 21 | 24 | 12 | 14 | 0.62 | 0.634 | 0.511-0.745 | 0.6 | 0.421-0.761 | 0.667 | 0.49-0.814 | FALSE | 0.006797813 | 1 | FALSE | -1.46806337 | 1.191801249 | 1 | 3.222727847 | FALSE |
| CALCOCO1 | 1.724225955 | 4.39014721 | 3.8266E-05 | 0.000267086 | TRUE | 23 | 25 | 11 | 12 | 0.637 | 0.676 | 0.555-0.782 | 0.657 | 0.478-0.809 | 0.694 | 0.519-0.837 | FALSE |  |  |  | -1.62422128 | -1.056408352 | 1 | 2.315275609 | FALSE |
| TSC22D3 | 1.011966692 | 4.37403004 | 4.0564E-05 | 0.000279847 | TRUE | 35 | 0 | 36 | 0 | 0.322 | 0.493 | 0.372-0.614 | 1 | 0.9-1 | 0 | 0-0.097 | FALSE |  |  |  | -1.26973744 | -1.383549976 | 0 | 1.103257968 | FALSE |
| FKBP5 | 1.07896911 | 4.31976269 | 4.9325E-05 | 0.000333027 | TRUE | 14 | 22 | 14 | 21 | 0.425 | 0.507 | 0.386-0.628 | 0.4 | 0.239-0.579 | 0.611 | 0.435-0.769 | FALSE |  |  |  | -1.40378241 | 2.3751938 | 1 | 1.478709222 | FALSE |
| HBEGF | 1.433829905 | 4.27355242 | 5.8207E-05 | 0.000387115 | TRUE | 7 | 33 | 3 | 28 | 0.409 | 0.563 | 0.44-0.681 | 0.2 | 0.084-0.369 | 0.917 | 0.775-0.982 | FALSE |  |  |  | -7.15683796 | -3.914691583 | 1 | 1.856790095 | FALSE |
| CC2D1A | 1.786879534 | 4.1898349 | 7.8383E-05 | 0.000496749 | TRUE | 20 | 25 | 11 | 15 | 0.59 | 0.634 | 0.511-0.745 | 0.571 | 0.394-0.737 | 0.694 | 0.519-0.837 | FALSE |  |  |  | -3.2481622 | 0.67259814 | 1 | 2.165815877 | FALSE |
| GLRX2 | 1.885316188 | 4.18225263 | 8.0513E-05 | 0.000507133 | TRUE | 29 | 16 | 20 | 6 | 0.578 | 0.634 | 0.511-0.745 | 0.829 | 0.664-0.934 | 0.444 | 0.279-0.619 | FALSE | 0.002260803 | 2 | FALSE | -1.5160435 | 0.243596766 | 1 | 3.023182148 | FALSE |
| MECR | 2.416351414 | 4.15953749 | 8.7232E-05 | 0.000538852 | TRUE | 35 | 0 | 36 | 0 | 0.329 | 0.493 | 0.372-0.614 | 1 | 0.9-1 | 0 | 0-0.097 | FALSE |  |  |  | -2.22376923 | -2.340646881 | 0 | 2.820279968 | FALSE |
| TNIP1 | 1.643832598 | 4.15822806 | 8.7635E-05 | 0.000538852 | TRUE | 3 | 36 | 0 | 32 | 0.475 | 0.549 | 0.427-0.668 | 0.086 | 0.018-0.231 | 1 | 0.903-1 | FALSE |  |  |  | -0.8237186 | -1.311124597 | 0 | 1.312771324 | TRUE |
| SMPD1 | 0.412373071 | 4.15643365 | 8.8191E-05 | 0.00053906 | FALSE | 26 | 18 | 18 | 9 | 0.591 | 0.62 | 0.497-0.732 | 0.743 | 0.567-0.875 | 0.5 | 0.329-0.671 | FALSE | 0.01025446 | 4 | TRUE | -4.23445568 | -6.386710024 | 0 | 0.394924811 | TRUE |
| C9orf40 | 1.273763219 | 4.15068663 | 8.9994E-05 | 0.000546843 | TRUE | 1 | 36 | 0 | 34 | 0.414 | 0.521 | 0.399-0.641 | 0.029 | 0.001-0.149 | 1 | 0.903-1 | FALSE |  |  |  | -4.03723412 | -1.963368381 | 1 | 1.216915246 | TRUE |
| CHCHD6 | 1.074379804 | 4.12854725 | 9.7276E-05 | 0.000584221 | TRUE | 8 | 35 | 1 | 27 | 0.607 | 0.606 | 0.483-0.72 | 0.229 | 0.104-0.401 | 0.972 | 0.855-0.999 | FALSE | 0.004609996 | 2 | FALSE | -0.42680477 | 0.833153548 | 1 | 1.810627225 | FALSE |
| ADGRE3 | 1.125047358 | 4.09072914 | 0.00011105 | 0.00065927 | TRUE | 32 | 10 | 26 | 3 | 0.571 | 0.592 | 0.468-0.707 | 0.914 | 0.769-0.982 | 0.278 | 0.142-0.452 | FALSE | 0.010152621 | 4 | TRUE | -3.72587724 | -1.044558736 | 1 | 1.689733474 | FALSE |
| SMURF1 | 1.18637177 | 4.02320045 | 0.00014044 | 0.000801526 | TRUE | 31 | 6 | 30 | 4 | 0.381 | 0.521 | 0.399-0.641 | 0.886 | 0.733-0.968 | 0.167 | 0.064-0.328 | FALSE |  |  |  | 0.30978158 | -0.015280632 | 0 | 1.498925715 | FALSE |
| BAIAP2 | 1.126611998 | 4.0163063 | 0.00014383 | 0.000816367 | TRUE | 30 | 9 | 27 | 5 | 0.475 | 0.549 | 0.427-0.668 | 0.857 | 0.697-0.952 | 0.25 | 0.121-0.422 | FALSE |  |  |  | -2.15159125 | -3.815168967 | 0 | 0.697970149 | TRUE |
| IL18 | 1.596600781 | 3.98882836 | 0.00015815 | 0.0008783 | TRUE | 3 | 33 | 3 | 32 | 0.304 | 0.507 | 0.386-0.628 | 0.086 | 0.018-0.231 | 0.917 | 0.775-0.982 | FALSE |  |  |  | -8.38089196 | -4.874551572 | 1 | 2.085843833 | FALSE |
| C4BPB | 1.148773653 | 3.98444796 | 0.00016055 | 0.000886886 | TRUE | 25 | 17 | 19 | 10 | 0.572 | 0.592 | 0.468-0.707 | 0.714 | 0.537-0.854 | 0.472 | 0.304-0.645 | FALSE | 0.012424924 | 1 | FALSE | -5.52927067 | -6.427294891 | 0 | 1.338663552 | FALSE |
| TNFRSF13B | 1.598631858 | 3.96509887 | 0.0001716 | 0.000932949 | TRUE | 6 | 33 | 3 | 29 | 0.408 | 0.549 | 0.427-0.668 | 0.171 | 0.066-0.336 | 0.917 | 0.775-0.982 | FALSE |  |  |  | -8.68306136 | -7.78145096 | 1 | 2.532714233 | FALSE |
| FYCO1 | 1.764729158 | 3.94255045 | 0.00018539 | 0.000997458 | TRUE | 29 | 17 | 19 | 6 | 0.606 | 0.648 | 0.525-0.758 | 0.829 | 0.664-0.934 | 0.472 | 0.304-0.645 | FALSE | 0.007869024 | 2 | FALSE | -3.03244155 | 1.385086545 | 1 | 2.647263614 | FALSE |
| USP8 | 1.018116087 | 3.92717932 | 0.0001954 | 0.001035842 | TRUE | 35 | 0 | 36 | 0 | 0.3 | 0.493 | 0.372-0.614 | 1 | 0.9-1 | 0 | 0-0.097 | FALSE |  |  |  | -3.07340012 | -3.735526784 | 0 | 1.707856674 | FALSE |
| CLEC4A | 1.35016129 | 3.9269784 | 0.00019554 | 0.001035842 | TRUE | 28 | 14 | 22 | 7 | 0.484 | 0.592 | 0.468-0.707 | 0.8 | 0.631-0.916 | 0.389 | 0.231-0.565 | FALSE | 0.007603737 | 1 | FALSE | -2.87606026 | 2.392754135 | 1 | 2.125137945 | FALSE |
| TNFRSF11B | 1.357238075 | 3.89722617 | 0.00021641 | 0.001134801 | TRUE | 5 | 33 | 3 | 30 | 0.433 | 0.535 | 0.413-0.655 | 0.143 | 0.048-0.303 | 0.917 | 0.775-0.982 | FALSE |  |  |  | -7.87360629 | -5.013978409 | 1 | 1.922978026 | FALSE |
| TRIP11 | 1.442220578 | 3.889144 | 0.00022244 | 0.0011547 | TRUE | 24 | 20 | 16 | 11 | 0.601 | 0.62 | 0.497-0.732 | 0.686 | 0.507-0.831 | 0.556 | 0.381-0.721 | FALSE | 0.009174523 | 4 | TRUE | -2.48743971 | 2.94712429 | 1 | 1.883865811 | FALSE |
| LRRN1 | 0.714141859 | 3.83688173 | 0.00026551 | 0.001317624 | FALSE | 19 | 28 | 8 | 16 | 0.61 | 0.662 | 0.54-0.77 | 0.543 | 0.366-0.712 | 0.778 | 0.608-0.899 | FALSE | 0.015565952 | 4 | TRUE | -4.04168345 | -5.697396292 | 0 | 0.835254796 | FALSE |
| TRIAP1 | 1.043276142 | 3.83580319 | 0.00026647 | 0.001317624 | TRUE | 32 | 5 | 31 | 3 | 0.307 | 0.521 | 0.399-0.641 | 0.914 | 0.769-0.982 | 0.139 | 0.047-0.295 | FALSE |  |  |  | -0.52795314 | 1.259393266 | 1 | 1.785519707 | FALSE |
| KHK | 2.076282569 | 3.82707244 | 0.00027443 | 0.001337211 | TRUE | 27 | 22 | 14 | 8 | 0.719 | 0.69 | 0.569-0.795 | 0.771 | 0.599-0.896 | 0.611 | 0.435-0.769 | TRUE | 0.01394312 | 4 | TRUE | -2.41950956 | -4.547641371 | 0 | 3.052720167 | FALSE |
| CD101 | 1.122555563 | 3.78940195 | 0.00031145 | 0.00148263 | TRUE | 27 | 19 | 17 | 8 | 0.626 | 0.648 | 0.525-0.758 | 0.771 | 0.599-0.896 | 0.528 | 0.355-0.696 | FALSE | 0.007832525 | 3 | TRUE | -4.32901132 | -4.026573989 | 1 | 2.042356499 | FALSE |
| GZMH | 2.090070561 | 3.77791864 | 0.00032366 | 0.001503331 | TRUE | 10 | 29 | 7 | 25 | 0.451 | 0.549 | 0.427-0.668 | 0.286 | 0.146-0.463 | 0.806 | 0.64-0.918 | FALSE | 0.002014005 | 1 | FALSE | -5.70356812 | -3.65968911 | 1 | 3.459945309 | FALSE |
| RBKS | 1.895232848 | 3.7710059 | 0.00033122 | 0.001520675 | TRUE | 29 | 11 | 25 | 6 | 0.484 | 0.563 | 0.44-0.681 | 0.829 | 0.664-0.934 | 0.306 | 0.163-0.481 | FALSE |  |  |  | -3.94571006 | -2.499002345 | 1 | 3.226344968 | FALSE |
| METAP1D | 2.042106182 | 3.75597574 | 0.00034826 | 0.001591815 | TRUE | 14 | 34 | 2 | 21 | 0.627 | 0.676 | 0.555-0.782 | 0.4 | 0.239-0.579 | 0.944 | 0.813-0.993 | FALSE | 0.00462456 | 2 | FALSE | -1.47999671 | -1.577241307 | 0 | 2.92186001 | FALSE |
| NOS1 | 1.417289817 | 3.7445151 | 0.00036181 | 0.001646459 | TRUE | 22 | 24 | 12 | 13 | 0.616 | 0.648 | 0.525-0.758 | 0.629 | 0.449-0.785 | 0.667 | 0.49-0.814 | FALSE | 0.006449107 | 3 | TRUE | -1.21660821 | -0.39535074 | 1 | 2.022265978 | FALSE |
| EVA1C | 1.293808245 | 3.70487869 | 0.00041265 | 0.001845314 | TRUE | 19 | 25 | 11 | 16 | 0.604 | 0.62 | 0.497-0.732 | 0.543 | 0.366-0.712 | 0.694 | 0.519-0.837 | FALSE | 0.006827078 | 3 | TRUE | -2.52184592 | 1.225932636 | 1 | 1.701916586 | FALSE |
| GALNT3 | 1.351882089 | 3.69800488 | 0.00042213 | 0.001879587 | TRUE | 1 | 36 | 0 | 34 | 0.374 | 0.521 | 0.399-0.641 | 0.029 | 0.001-0.149 | 1 | 0.903-1 | FALSE |  |  |  | -3.05751169 | -1.083402678 | 1 | 2.525701519 | FALSE |
| ACAA1 | -1.028328677 | -3.69546983 | 0.00042568 | 0.001887257 | FALSE | 26 | 27 | 9 | 9 | 0.791 | 0.746 | 0.629-0.842 | 0.743 | 0.567-0.875 | 0.75 | 0.578-0.879 | TRUE |  |  |  | -3.48247729 | -9.407851034 | 0 | 1.047238961 | FALSE |
| RGS8 | -0.476446144 | -3.6760439 | 0.00045384 | 0.001998276 | FALSE | 25 | 13 | 23 | 10 | 0.462 | 0.535 | 0.413-0.655 | 0.714 | 0.537-0.854 | 0.361 | 0.208-0.538 | FALSE | 0.012100498 | 3 | TRUE | 0.1716063 | -2.268202905 | 0 | 0.53481575 | FALSE |
| PRELP | 0.553595393 | 3.67061424 | 0.00046203 | 0.002014047 | FALSE | 35 | 10 | 26 | 0 | 0.622 | 0.634 | 0.511-0.745 | 1 | 0.9-1 | 0.278 | 0.142-0.452 | FALSE | 0.009017581 | 3 | TRUE | -3.91278914 | -5.779829236 | 0 | 0.537623588 | TRUE |
| BCL2L15 | 1.059431077 | 3.66513087 | 0.00047043 | 0.002030377 | TRUE | 30 | 6 | 30 | 5 | 0.331 | 0.507 | 0.386-0.628 | 0.857 | 0.697-0.952 | 0.167 | 0.064-0.328 | FALSE |  |  |  | -3.59660442 | -1.269915637 | 1 | 2.079580005 | FALSE |
| TRIM25 | 2.127566838 | 3.65430404 | 0.00048746 | 0.00208078 | TRUE | 19 | 27 | 9 | 16 | 0.617 | 0.648 | 0.525-0.758 | 0.543 | 0.366-0.712 | 0.75 | 0.578-0.879 | FALSE | 0.012680107 | 3 | TRUE | -4.56443282 | -6.800984331 | 0 | 2.504045628 | FALSE |
| ITGB6 | 1.191590814 | 3.63650132 | 0.00051674 | 0.002178765 | TRUE | 20 | 25 | 11 | 15 | 0.619 | 0.634 | 0.511-0.745 | 0.571 | 0.394-0.737 | 0.694 | 0.519-0.837 | FALSE | 0.011404155 | 2 | FALSE | -1.96105461 | 0.664246523 | 1 | 1.520976156 | FALSE |
| CTH | 2.035968756 | 3.58601511 | 0.00060916 | 0.002527166 | TRUE | 25 | 17 | 19 | 10 | 0.589 | 0.592 | 0.468-0.707 | 0.714 | 0.537-0.854 | 0.472 | 0.304-0.645 | FALSE | 0.004247872 | 1 | FALSE | -3.03212425 | -4.759680188 | 0 | 2.366283011 | FALSE |
| SETMAR | 0.749806944 | 3.54744788 | 0.00069012 | 0.002782676 | FALSE | 24 | 26 | 10 | 11 | 0.714 | 0.704 | 0.584-0.807 | 0.686 | 0.507-0.831 | 0.722 | 0.548-0.858 | TRUE | 0.033132699 | 4 | TRUE | -2.76592454 | -4.505421468 | 0 | 0.540142975 | TRUE |
| PGLYRP3 | 1.181886376 | 3.52909989 | 0.00073213 | 0.002920045 | TRUE | 26 | 21 | 15 | 9 | 0.626 | 0.662 | 0.54-0.77 | 0.743 | 0.567-0.875 | 0.583 | 0.408-0.745 | FALSE | 0.01963245 | 3 | TRUE | -2.08629599 | 2.727892515 | 1 | 1.597826475 | FALSE |
| EREG | 1.273375416 | 3.52443057 | 0.0007432 | 0.002941487 | TRUE | 35 | 0 | 36 | 0 | 0.367 | 0.493 | 0.372-0.614 | 1 | 0.9-1 | 0 | 0-0.097 | FALSE |  |  |  | -3.32589651 | 1.954891575 | 1 | 1.945514822 | FALSE |
| CRADD | 1.440445929 | 3.51385889 | 0.00076886 | 0.003031411 | TRUE | 35 | 1 | 35 | 0 | 0.289 | 0.507 | 0.386-0.628 | 1 | 0.9-1 | 0.028 | 0.001-0.145 | FALSE |  |  |  | -3.32247876 | -2.508904086 | 1 | 2.064347542 | FALSE |
| MAP2K6 | 0.87295868 | 3.4926902 | 0.00082277 | 0.003183239 | FALSE | 17 | 28 | 8 | 18 | 0.629 | 0.634 | 0.511-0.745 | 0.486 | 0.314-0.66 | 0.778 | 0.608-0.899 | FALSE | 0.028268607 | 4 | TRUE | -0.26478195 | -0.215140096 | 1 | 1.290466482 | FALSE |
| NUB1 | 1.190891507 | 3.43902396 | 0.00097595 | 0.003600566 | TRUE | 8 | 33 | 3 | 27 | 0.494 | 0.577 | 0.454-0.694 | 0.229 | 0.104-0.401 | 0.917 | 0.775-0.982 | FALSE |  |  |  | -0.59249595 | -0.133363543 | 1 | 1.608401666 | FALSE |
| SWAP70 | 1.394396694 | 3.41762598 | 0.00104425 | 0.00381168 | TRUE | 30 | 8 | 28 | 5 | 0.436 | 0.535 | 0.413-0.655 | 0.857 | 0.697-0.952 | 0.222 | 0.101-0.392 | FALSE |  |  |  | -3.88681665 | -3.622067565 | 1 | 2.744821432 | FALSE |
| DAPK2 | 1.918671886 | 3.4146441 | 0.00105411 | 0.00383415 | TRUE | 15 | 32 | 4 | 20 | 0.623 | 0.662 | 0.54-0.77 | 0.429 | 0.263-0.606 | 0.889 | 0.739-0.969 | FALSE | 0.007501363 | 1 | FALSE | -5.85745697 | -7.874900917 | 0 | 2.814264541 | FALSE |
| CEACAM21 | 1.575485342 | 3.34656072 | 0.00130487 | 0.00460044 | TRUE | 2 | 36 | 0 | 33 | 0.417 | 0.535 | 0.413-0.655 | 0.057 | 0.007-0.192 | 1 | 0.903-1 | FALSE |  |  |  | -3.52317217 | -2.339583747 | 1 | 2.814844493 | FALSE |
| DCPS | 1.531195828 | 3.34345106 | 0.00131757 | 0.004629413 | TRUE | 26 | 12 | 24 | 9 | 0.498 | 0.535 | 0.413-0.655 | 0.743 | 0.567-0.875 | 0.333 | 0.186-0.51 | FALSE |  |  |  | -5.11543831 | -4.318082306 | 1 | 3.738387711 | FALSE |
| CD33 | 1.018052345 | 3.27075267 | 0.0016498 | 0.005643177 | TRUE | 12 | 29 | 7 | 23 | 0.493 | 0.577 | 0.454-0.694 | 0.343 | 0.191-0.522 | 0.806 | 0.64-0.918 | FALSE |  |  |  | -7.37797862 | -3.358048905 | 1 | 2.216170627 | FALSE |
| CNPY4 | 0.443816192 | 3.2664156 | 0.00167192 | 0.005699972 | FALSE | 18 | 23 | 13 | 17 | 0.543 | 0.577 | 0.454-0.694 | 0.514 | 0.34-0.686 | 0.639 | 0.462-0.792 | FALSE | 0.016999641 | 4 | TRUE | -1.89172018 | -3.87430947 | 0 | 0.721573165 | FALSE |
| EIF2S2 | -0.971793228 | -3.23839773 | 0.00182168 | 0.006109727 | FALSE | 25 | 27 | 9 | 10 | 0.769 | 0.732 | 0.614-0.831 | 0.714 | 0.537-0.854 | 0.75 | 0.578-0.879 | TRUE |  |  |  | -2.09018517 | -5.403571667 | 0 | 1.619238694 | FALSE |
| PLA2G10 | 1.152038341 | 3.14983642 | 0.00238198 | 0.00752472 | TRUE | 28 | 9 | 27 | 7 | 0.47 | 0.521 | 0.399-0.641 | 0.8 | 0.631-0.916 | 0.25 | 0.121-0.422 | FALSE |  |  |  | -4.75370304 | -6.538280544 | 0 | 1.282303093 | FALSE |
| NOS2 | 1.451438969 | 3.14089637 | 0.00244672 | 0.007682246 | TRUE | 15 | 30 | 6 | 20 | 0.61 | 0.634 | 0.511-0.745 | 0.429 | 0.263-0.606 | 0.833 | 0.672-0.936 | FALSE | 0.007579692 | 2 | FALSE | -0.63049472 | -2.164323417 | 0 | 1.586409137 | FALSE |
| GZMB | 1.097116629 | 3.13949391 | 0.00245702 | 0.00769122 | TRUE | 27 | 10 | 26 | 8 | 0.379 | 0.521 | 0.399-0.641 | 0.771 | 0.599-0.896 | 0.278 | 0.142-0.452 | FALSE |  |  |  | -0.66669514 | 2.336216683 | 1 | 2.175642634 | FALSE |
| RPH3A | 1.932187773 | 3.12692641 | 0.00255118 | 0.007914021 | TRUE | 28 | 15 | 21 | 7 | 0.574 | 0.606 | 0.483-0.72 | 0.8 | 0.631-0.916 | 0.417 | 0.255-0.592 | FALSE |  |  |  | -4.9178258 | 0.00831436 | 1 | 5.203073976 | FALSE |
| DPY30 | 1.128186851 | 3.10857766 | 0.00269472 | 0.008235633 | TRUE | 35 | 0 | 36 | 0 | 0.34 | 0.493 | 0.372-0.614 | 1 | 0.9-1 | 0 | 0-0.097 | FALSE |  |  |  | -2.83664849 | -4.008085037 | 0 | 1.559026419 | FALSE |
| RBFOX3 | 1.063597744 | 3.10460268 | 0.00272679 | 0.008309071 | TRUE | 25 | 19 | 17 | 10 | 0.612 | 0.62 | 0.497-0.732 | 0.714 | 0.537-0.854 | 0.528 | 0.355-0.696 | FALSE |  |  |  | -1.96807922 | 2.884852262 | 1 | 2.11433698 | FALSE |
| INPP1 | 1.094049852 | 3.10318213 | 0.00273834 | 0.008319718 | TRUE | 30 | 14 | 22 | 5 | 0.587 | 0.62 | 0.497-0.732 | 0.857 | 0.697-0.952 | 0.389 | 0.231-0.565 | FALSE | 0.017116408 | 4 | TRUE | -1.69205837 | 0.35766774 | 1 | 2.487353675 | FALSE |
| MELTF | 1.054514764 | 3.08477234 | 0.00289218 | 0.008710268 | TRUE | 35 | 2 | 34 | 0 | 0.413 | 0.521 | 0.399-0.641 | 1 | 0.9-1 | 0.056 | 0.007-0.187 | FALSE |  |  |  | -8.06841523 | -4.338828691 | 1 | 1.42647576 | FALSE |
| MMP10 | 1.529922189 | 3.08285567 | 0.00290865 | 0.00873441 | TRUE | 28 | 17 | 19 | 7 | 0.584 | 0.634 | 0.511-0.745 | 0.8 | 0.631-0.916 | 0.472 | 0.304-0.645 | FALSE |  |  |  | -8.92880093 | -4.009121933 | 1 | 3.542973658 | FALSE |
| KYNU | 1.703510161 | 3.04518593 | 0.00325065 | 0.009677011 | TRUE | 26 | 14 | 22 | 9 | 0.464 | 0.563 | 0.44-0.681 | 0.743 | 0.567-0.875 | 0.389 | 0.231-0.565 | FALSE |  |  |  | -2.494554 | -3.72153942 | 0 | 3.008253898 | FALSE |
| RC3H1 | 1.639360937 | 3.00817191 | 0.00362282 | 0.010571662 | TRUE | 6 | 35 | 1 | 29 | 0.397 | 0.577 | 0.454-0.694 | 0.171 | 0.066-0.336 | 0.972 | 0.855-0.999 | FALSE |  |  |  | -2.92841 | -4.915190287 | 0 | 3.212481053 | FALSE |
| ACP6 | 1.259972008 | 2.99232408 | 0.00379395 | 0.011039857 | TRUE | 30 | 15 | 21 | 5 | 0.625 | 0.634 | 0.511-0.745 | 0.857 | 0.697-0.952 | 0.417 | 0.255-0.592 | FALSE | 0.009516717 | 3 | TRUE | -4.66400095 | -4.652340939 | 1 | 2.507991364 | FALSE |
| CXCL8 | 1.103017186 | 2.98753698 | 0.0038471 | 0.011131803 | TRUE | 4 | 34 | 2 | 31 | 0.429 | 0.535 | 0.413-0.655 | 0.114 | 0.032-0.267 | 0.944 | 0.813-0.993 | FALSE |  |  |  | -4.14589081 | 6.053299275 | 1 | 2.309766794 | FALSE |
| CCN4 | 0.930942821 | 2.98460392 | 0.00388 | 0.011172095 | FALSE | 28 | 14 | 22 | 7 | 0.534 | 0.592 | 0.468-0.707 | 0.8 | 0.631-0.916 | 0.389 | 0.231-0.565 | FALSE | 0.007713738 | 3 | TRUE | -7.20319871 | -8.065403505 | 0 | 1.515767129 | FALSE |
| CXCL17 | -0.827953152 | -2.96036526 | 0.00416209 | 0.011805083 | FALSE | 18 | 33 | 3 | 17 | 0.707 | 0.718 | 0.599-0.819 | 0.514 | 0.34-0.686 | 0.917 | 0.775-0.982 | TRUE |  |  |  | -3.16009692 | -6.007630071 | 0 | 1.353159467 | FALSE |
| SH3PXD2B | 1.454414354 | 2.9136553 | 0.00475998 | 0.013120956 | TRUE | 22 | 24 | 12 | 13 | 0.687 | 0.648 | 0.525-0.758 | 0.629 | 0.449-0.785 | 0.667 | 0.49-0.814 | FALSE | 0.022739037 | 5 | TRUE | -2.34218561 | 1.958500137 | 1 | 2.914414247 | FALSE |
| PGM2 | 1.05218327 | 2.90379533 | 0.00489593 | 0.013450788 | TRUE | 23 | 20 | 16 | 12 | 0.585 | 0.606 | 0.483-0.72 | 0.657 | 0.478-0.809 | 0.556 | 0.381-0.721 | FALSE | 0.008096614 | 2 | FALSE | -0.27293961 | 2.027994029 | 1 | 2.711414974 | FALSE |
| RIGI | 1.350969721 | 2.86888264 | 0.00540665 | 0.014558537 | TRUE | 3 | 36 | 0 | 32 | 0.453 | 0.549 | 0.427-0.668 | 0.086 | 0.018-0.231 | 1 | 0.903-1 | FALSE |  |  |  | -2.19425033 | -2.438568498 | 0 | 2.999116317 | FALSE |
| AMIGO2 | -0.506325629 | -2.85685654 | 0.00559365 | 0.015008415 | FALSE | 27 | 13 | 23 | 8 | 0.509 | 0.563 | 0.44-0.681 | 0.771 | 0.599-0.896 | 0.361 | 0.208-0.538 | FALSE | 0.008504988 | 3 | TRUE | -5.9222551 | -9.333305333 | 0 | 0.971301022 | FALSE |
| S100A13 | 1.040498661 | 2.7355497 | 0.00784197 | 0.019982641 | TRUE | 2 | 35 | 1 | 33 | 0.394 | 0.521 | 0.399-0.641 | 0.057 | 0.007-0.192 | 0.972 | 0.855-0.999 | FALSE |  |  |  | -5.75835889 | 3.747230025 | 1 | 1.560313892 | FALSE |
| IL2 | -0.576284614 | -2.73072847 | 0.00794643 | 0.020168705 | FALSE | 20 | 21 | 15 | 15 | 0.552 | 0.577 | 0.454-0.694 | 0.571 | 0.394-0.737 | 0.583 | 0.408-0.745 | FALSE | 0.014990638 | 4 | TRUE | -0.10031503 | -3.208504547 | 0 | 0.854342529 | FALSE |
| FSTL1 | -0.220622987 | -2.70098082 | 0.00861963 | 0.021507444 | FALSE | 12 | 29 | 7 | 23 | 0.566 | 0.577 | 0.454-0.694 | 0.343 | 0.191-0.522 | 0.806 | 0.64-0.918 | FALSE | 0.016200885 | 4 | TRUE | -0.85963157 | -2.796821318 | 0 | 0.421038961 | FALSE |
| ACP3 | 1.420005541 | 2.69534435 | 0.00875291 | 0.021720871 | TRUE | 23 | 14 | 22 | 12 | 0.461 | 0.521 | 0.399-0.641 | 0.657 | 0.478-0.809 | 0.389 | 0.231-0.565 | FALSE |  |  |  | -4.17831705 | -3.72124067 | 1 | 2.859995168 | FALSE |
| LMOD1 | -0.781448615 | -2.69245869 | 0.00882187 | 0.021749377 | FALSE | 33 | 3 | 33 | 2 | 0.383 | 0.507 | 0.386-0.628 | 0.943 | 0.808-0.993 | 0.083 | 0.018-0.225 | FALSE | 0.015153473 | 4 | TRUE | -3.33288931 | -9.234199079 | 0 | 1.314676305 | FALSE |
| UBE2Z | -0.462973784 | -2.68864309 | 0.00891381 | 0.021923722 | FALSE | 2 | 35 | 1 | 33 | 0.383 | 0.521 | 0.399-0.641 | 0.057 | 0.007-0.192 | 0.972 | 0.855-0.999 | FALSE | 0.004405583 | 3 | TRUE | -0.30609545 | -2.568227298 | 0 | 0.624812613 | FALSE |
| CD5 | 0.505201649 | 2.66355456 | 0.00954042 | 0.023353677 | FALSE | 13 | 33 | 3 | 22 | 0.598 | 0.648 | 0.525-0.758 | 0.371 | 0.215-0.551 | 0.917 | 0.775-0.982 | FALSE | 0.005110533 | 3 | TRUE | -2.65286083 | -3.273702999 | 0 | 0.887830374 | FALSE |
| AFAP1L1 | 1.233083742 | 2.60128281 | 0.01127264 | 0.026830963 | TRUE | 26 | 16 | 20 | 9 | 0.583 | 0.592 | 0.468-0.707 | 0.743 | 0.567-0.875 | 0.444 | 0.279-0.619 | FALSE | 0.010969646 | 1 | FALSE | -1.62090669 | -5.63415599 | 0 | 2.25855641 | FALSE |
| NIT1 | 1.89686225 | 2.5648471 | 0.01241385 | 0.029078243 | TRUE | 33 | 4 | 32 | 2 | 0.344 | 0.521 | 0.399-0.641 | 0.943 | 0.808-0.993 | 0.111 | 0.031-0.261 | FALSE |  |  |  | -5.82792509 | -4.585461421 | 1 | 4.540626101 | FALSE |
| ERBB4 | 0.435425643 | 2.51491653 | 0.01414737 | 0.032476065 | FALSE | 6 | 36 | 0 | 29 | 0.475 | 0.592 | 0.468-0.707 | 0.171 | 0.066-0.336 | 1 | 0.903-1 | FALSE | 0.004668749 | 4 | TRUE | -5.05635721 | -6.865158731 | 0 | 0.598773801 | FALSE |
| ACY3 | 1.393667608 | 2.49862869 | 0.01475834 | 0.033654225 | TRUE | 34 | 2 | 34 | 1 | 0.283 | 0.507 | 0.386-0.628 | 0.971 | 0.851-0.999 | 0.056 | 0.007-0.187 | FALSE |  |  |  | -6.42547583 | -7.389726947 | 0 | 3.237307519 | FALSE |
| IL1A | 0.588155929 | 2.47680508 | 0.01561416 | 0.035113871 | FALSE | 19 | 25 | 11 | 16 | 0.583 | 0.62 | 0.497-0.732 | 0.543 | 0.366-0.712 | 0.694 | 0.519-0.837 | FALSE | 0.010439205 | 4 | TRUE | 1.40530113 | 10.29016921 | 1 | 1.376795566 | FALSE |
| GZMA | 1.235777956 | 2.47517026 | 0.01568003 | 0.035135506 | TRUE | 35 | 3 | 33 | 0 | 0.483 | 0.535 | 0.413-0.655 | 1 | 0.9-1 | 0.083 | 0.018-0.225 | FALSE |  |  |  | -7.89433581 | -2.109242465 | 1 | 2.530825176 | FALSE |
| SIGLEC6 | 1.024418866 | 2.45954707 | 0.01632222 | 0.036104609 | TRUE | 35 | 1 | 35 | 0 | 0.29 | 0.507 | 0.386-0.628 | 1 | 0.9-1 | 0.028 | 0.001-0.145 | FALSE |  |  |  | -6.82878716 | -9.24085817 | 0 | 2.123183259 | FALSE |
| ICA1 | -0.214324323 | -2.37903859 | 0.02002024 | 0.043265492 | FALSE | 16 | 34 | 2 | 19 | 0.729 | 0.704 | 0.584-0.807 | 0.457 | 0.288-0.634 | 0.944 | 0.813-0.993 | TRUE |  |  |  | -0.35445972 | -1.841014927 | 0 | 0.190321873 | FALSE |
| CCL8 | 1.035108357 | 2.31143671 | 0.0236838 | 0.049525036 | TRUE | 35 | 1 | 35 | 0 | 0.34 | 0.507 | 0.386-0.628 | 1 | 0.9-1 | 0.028 | 0.001-0.145 | FALSE |  |  |  | -7.27451437 | -11.69380749 | 0 | 2.334679517 | FALSE |
| PCBP2 | -0.398802243 | -2.25841233 | 0.02696092 | 0.055259183 | FALSE | 19 | 29 | 7 | 16 | 0.702 | 0.676 | 0.555-0.782 | 0.543 | 0.366-0.712 | 0.806 | 0.64-0.918 | TRUE |  |  |  | -1.82106872 | -5.151842332 | 0 | 0.66775171 | FALSE |
| MAG | 0.303820488 | 2.22065433 | 0.02953209 | 0.05900706 | FALSE | 31 | 12 | 24 | 4 | 0.59 | 0.606 | 0.483-0.72 | 0.886 | 0.733-0.968 | 0.333 | 0.186-0.51 | FALSE | 0.01581268 | 4 | TRUE | -0.19640438 | -1.740650352 | 0 | 0.612651464 | FALSE |
| DDX39A | 0.218998358 | 2.09081383 | 0.04008679 | 0.076163993 | FALSE | 29 | 12 | 24 | 6 | 0.514 | 0.577 | 0.454-0.694 | 0.829 | 0.664-0.934 | 0.333 | 0.186-0.51 | FALSE | 0.015687331 | 4 | TRUE | -9.0404E-05 | -0.484175453 | 0 | 0.455475595 | FALSE |
| C8orf34 | 0.309741327 | 2.06209894 | 0.04282011 | 0.079843281 | FALSE | 6 | 34 | 2 | 29 | 0.536 | 0.563 | 0.44-0.681 | 0.171 | 0.066-0.336 | 0.944 | 0.813-0.993 | FALSE | 0.011027735 | 3 | TRUE | 0.80462077 | -0.488928471 | 0 | 0.31596177 | FALSE |
| DNM1 | -0.525427717 | -1.97974103 | 0.05156859 | 0.094074171 | FALSE | 18 | 30 | 6 | 17 | 0.714 | 0.676 | 0.555-0.782 | 0.514 | 0.34-0.686 | 0.833 | 0.672-0.936 | TRUE |  |  |  | -3.26524693 | -8.341093545 | 0 | 1.269922071 | FALSE |
| FGF5 | -0.182808646 | -1.97082281 | 0.0526018 | 0.095595129 | FALSE | 17 | 27 | 9 | 18 | 0.594 | 0.62 | 0.497-0.732 | 0.486 | 0.314-0.66 | 0.75 | 0.578-0.879 | FALSE | 0.030546357 | 4 | TRUE | 0.13858861 | 0.02546597 | 0 | 0.270758 | FALSE |
| PSME1 | -0.502122645 | -1.92147124 | 0.05864411 | 0.104808587 | FALSE | 21 | 34 | 2 | 14 | 0.762 | 0.775 | 0.66-0.865 | 0.6 | 0.421-0.761 | 0.944 | 0.813-0.993 | TRUE |  |  |  | -3.2259415 | -3.154932964 | 1 | 1.916308736 | FALSE |
| RFC4 | 0.501906523 | 1.88772383 | 0.06310656 | 0.112201508 | FALSE | 21 | 25 | 11 | 14 | 0.619 | 0.648 | 0.525-0.758 | 0.6 | 0.421-0.761 | 0.694 | 0.519-0.837 | FALSE | 0.014769865 | 4 | TRUE | -2.4356027 | -3.052882058 | 0 | 1.032376606 | FALSE |
| CASP4 | -0.330836701 | -1.87626353 | 0.06468574 | 0.114099971 | FALSE | 12 | 30 | 6 | 23 | 0.54 | 0.592 | 0.468-0.707 | 0.343 | 0.191-0.522 | 0.833 | 0.672-0.936 | FALSE | 0.021924934 | 4 | TRUE | -2.84753422 | -6.483939344 | 0 | 0.908262242 | FALSE |
| SCG2 | 0.168958359 | 1.87597011 | 0.0647266 | 0.114099971 | FALSE | 23 | 21 | 15 | 12 | 0.604 | 0.62 | 0.497-0.732 | 0.657 | 0.478-0.809 | 0.583 | 0.408-0.745 | FALSE | 0.019986703 | 4 | TRUE | -2.37021417 | -3.450483608 | 0 | 0.335937388 | FALSE |
| SLA2 | -0.16952725 | -1.82932675 | 0.07150494 | 0.122902833 | FALSE | 22 | 23 | 13 | 13 | 0.536 | 0.634 | 0.511-0.745 | 0.629 | 0.449-0.785 | 0.639 | 0.462-0.792 | FALSE | 0.013293017 | 3 | TRUE | 0.09445529 | -1.100486675 | 0 | 0.387973548 | FALSE |
| CD226 | -0.318016027 | -1.81710809 | 0.0733757 | 0.125700005 | FALSE | 30 | 13 | 23 | 5 | 0.57 | 0.606 | 0.483-0.72 | 0.857 | 0.697-0.952 | 0.361 | 0.208-0.538 | FALSE | 0.013972408 | 4 | TRUE | -1.81239761 | -5.319935075 | 0 | 0.779809956 | FALSE |
| PARP1 | -0.094480047 | -1.78062942 | 0.07920545 | 0.131542173 | FALSE | 22 | 28 | 8 | 13 | 0.702 | 0.704 | 0.584-0.807 | 0.629 | 0.449-0.785 | 0.778 | 0.608-0.899 | TRUE |  |  |  | -0.31850208 | -1.038640967 | 0 | 0.200976814 | FALSE |
| TLR4 | 0.3054061 | 1.70974722 | 0.09163363 | 0.148133859 | FALSE | 20 | 26 | 10 | 15 | 0.646 | 0.648 | 0.525-0.758 | 0.571 | 0.394-0.737 | 0.722 | 0.548-0.858 | FALSE | 0.015991399 | 3 | TRUE | -0.67465327 | -1.281771146 | 0 | 0.817291791 | FALSE |
| CCL11 | 0.17924057 | 1.60456064 | 0.11298284 | 0.1784576 | FALSE | 7 | 32 | 4 | 28 | 0.408 | 0.549 | 0.427-0.668 | 0.2 | 0.084-0.369 | 0.889 | 0.739-0.969 | FALSE | 0.001814681 | 3 | TRUE | -3.99578268 | -7.084772168 | 0 | 0.493832551 | FALSE |
| ZC3H12D | 0.482694271 | 1.58391081 | 0.11761141 | 0.184079682 | FALSE | 34 | 10 | 26 | 1 | 0.585 | 0.62 | 0.497-0.732 | 0.971 | 0.851-0.999 | 0.278 | 0.142-0.452 | FALSE | 0.016485592 | 4 | TRUE | -0.92797306 | -3.185013734 | 0 | 1.245577931 | FALSE |
| NACC1 | 0.322130998 | 1.48051697 | 0.14311261 | 0.214565059 | FALSE | 17 | 29 | 7 | 18 | 0.626 | 0.648 | 0.525-0.758 | 0.486 | 0.314-0.66 | 0.806 | 0.64-0.918 | FALSE | 0.01410914 | 4 | TRUE | -1.04398468 | -3.641614308 | 0 | 0.614527632 | FALSE |
| NRTN | -0.181722814 | -1.39385299 | 0.16766342 | 0.242571864 | FALSE | 21 | 19 | 17 | 14 | 0.476 | 0.563 | 0.44-0.681 | 0.6 | 0.421-0.761 | 0.528 | 0.355-0.696 | FALSE | 0.008404923 | 3 | TRUE | -0.83662509 | -3.484294873 | 0 | 0.575853221 | FALSE |
| SEZ6L2 | -0.174626608 | -1.18132679 | 0.24137317 | 0.334233888 | FALSE | 28 | 13 | 23 | 7 | 0.492 | 0.577 | 0.454-0.694 | 0.8 | 0.631-0.916 | 0.361 | 0.208-0.538 | FALSE | 0.017618128 | 4 | TRUE | -1.58548829 | -4.35850358 | 0 | 1.014583585 | FALSE |
| LAYN | -0.27518145 | -1.03444889 | 0.30439986 | 0.402320595 | FALSE | 30 | 12 | 24 | 5 | 0.517 | 0.592 | 0.468-0.707 | 0.857 | 0.697-0.952 | 0.333 | 0.186-0.51 | FALSE | 0.007737694 | 3 | TRUE | -1.56466865 | -2.337587106 | 0 | 1.488304842 | FALSE |
| IFNL4 | -0.17746857 | -0.98212082 | 0.32933996 | 0.429014095 | FALSE | 30 | 14 | 22 | 5 | 0.575 | 0.62 | 0.497-0.732 | 0.857 | 0.697-0.952 | 0.389 | 0.231-0.565 | FALSE | 0.017508327 | 4 | TRUE | -3.07556287 | -6.223845093 | 0 | 0.833234878 | FALSE |
| WFDC3 | -0.164519432 | -0.8112917 | 0.41988092 | 0.518824147 | FALSE | 32 | 4 | 32 | 3 | 0.387 | 0.507 | 0.386-0.628 | 0.914 | 0.769-0.982 | 0.111 | 0.031-0.261 | FALSE | 0.005606617 | 3 | TRUE | -1.56209641 | -4.04615434 | 0 | 0.93700286 | FALSE |
| BACE1 | -0.277435452 | -0.80310731 | 0.42456475 | 0.522735862 | FALSE | 27 | 12 | 24 | 8 | 0.451 | 0.549 | 0.427-0.668 | 0.771 | 0.599-0.896 | 0.333 | 0.186-0.51 | FALSE | 0.010847236 | 3 | TRUE | -3.10464265 | -7.100477309 | 0 | 1.703790959 | FALSE |
| GPC1 | 0.231706978 | 0.70446291 | 0.48342451 | 0.581347522 | FALSE | 19 | 25 | 11 | 16 | 0.601 | 0.62 | 0.497-0.732 | 0.543 | 0.366-0.712 | 0.694 | 0.519-0.837 | FALSE | 0.011312237 | 4 | TRUE | -5.01889179 | -6.129690121 | 0 | 1.4114667 | FALSE |
| GFRA1 | 0.194100872 | 0.69846929 | 0.48714114 | 0.584456205 | FALSE | 20 | 21 | 15 | 15 | 0.54 | 0.577 | 0.454-0.694 | 0.571 | 0.394-0.737 | 0.583 | 0.408-0.745 | FALSE | 0.007764372 | 3 | TRUE | -6.42981182 | -9.653565432 | 0 | 1.323643241 | FALSE |
| SMAD3 | 0.039885161 | 0.58137147 | 0.56281182 | 0.656931767 | FALSE | 35 | 10 | 26 | 0 | 0.613 | 0.634 | 0.511-0.745 | 1 | 0.9-1 | 0.278 | 0.142-0.452 | FALSE | 0.017682787 | 3 | TRUE | 0.1038527 | -0.291964641 | 0 | 0.229390678 | FALSE |
| DTX3 | 0.091822987 | 0.56296258 | 0.57521507 | 0.669140948 | FALSE | 4 | 35 | 1 | 31 | 0.477 | 0.549 | 0.427-0.668 | 0.114 | 0.032-0.267 | 0.972 | 0.855-0.999 | FALSE | 0.008229757 | 4 | TRUE | -0.73282579 | -1.280555516 | 0 | 0.612751915 | FALSE |
| LPO | -0.051885185 | -0.47995037 | 0.63272238 | 0.721415248 | FALSE | 15 | 32 | 4 | 20 | 0.62 | 0.662 | 0.54-0.77 | 0.429 | 0.263-0.606 | 0.889 | 0.739-0.969 | FALSE | 0.027563164 | 4 | TRUE | -3.64587888 | 7.123835567 | 1 | 0.448892601 | FALSE |
| CTSF | 0.125968636 | 0.4562562 | 0.64958362 | 0.736017112 | FALSE | 11 | 29 | 7 | 24 | 0.521 | 0.563 | 0.44-0.681 | 0.314 | 0.169-0.493 | 0.806 | 0.64-0.918 | FALSE | 0.006490596 | 3 | TRUE | -3.84049036 | -7.374428111 | 0 | 0.5306496 | FALSE |
| PRTG | 0.056620191 | 0.39585725 | 0.69338483 | 0.773505967 | FALSE | 33 | 6 | 30 | 2 | 0.475 | 0.549 | 0.427-0.668 | 0.943 | 0.808-0.993 | 0.167 | 0.064-0.328 | FALSE | 0.011060904 | 3 | TRUE | -3.50736806 | -6.473988356 | 0 | 0.427470384 | FALSE |
| OBP2B | 0.093697527 | 0.39327932 | 0.69527917 | 0.774384605 | FALSE | 6 | 34 | 2 | 29 | 0.498 | 0.563 | 0.44-0.681 | 0.171 | 0.066-0.336 | 0.944 | 0.813-0.993 | FALSE | 0.003797623 | 3 | TRUE | -4.36596137 | -7.408554323 | 0 | 0.843876391 | FALSE |
| NCAN | -0.081946624 | -0.3279819 | 0.74388037 | 0.810578507 | FALSE | 16 | 23 | 13 | 19 | 0.505 | 0.549 | 0.427-0.668 | 0.457 | 0.288-0.634 | 0.639 | 0.462-0.792 | FALSE | 0.012547783 | 4 | TRUE | -0.92395376 | -8.21521415 | 0 | 1.619262032 | FALSE |
| `HLA-A` | 0.068874235 | 0.30105127 | 0.76424597 | 0.830143094 | FALSE | 14 | 30 | 6 | 21 | 0.596 | 0.62 | 0.497-0.732 | 0.4 | 0.239-0.579 | 0.833 | 0.672-0.936 | FALSE | 0.021116227 | 4 | TRUE | -1.96640879 | -3.4430042 | 0 | 1.379743908 | FALSE |
| PTPN14 | -0.074919132 | -0.24042163 | 0.81068892 | 0.869664753 | FALSE | 19 | 17 | 19 | 16 | 0.444 | 0.507 | 0.386-0.628 | 0.543 | 0.366-0.712 | 0.472 | 0.304-0.645 | FALSE | 0.003526564 | 3 | TRUE | -0.8070168 | -4.140409232 | 0 | 1.128855171 | FALSE |
| UBOX5 | -0.015865054 | -0.20910308 | 0.83496021 | 0.891026754 | FALSE | 35 | 4 | 32 | 0 | 0.477 | 0.549 | 0.427-0.668 | 1 | 0.9-1 | 0.111 | 0.031-0.261 | FALSE | 0.007743395 | 3 | TRUE | 0.33056799 | -0.986716225 | 0 | 0.407282503 | FALSE |
| PRKAB1 | -0.05539794 | -0.16240435 | 0.87144388 | 0.916702172 | FALSE | 16 | 30 | 6 | 19 | 0.626 | 0.648 | 0.525-0.758 | 0.457 | 0.288-0.634 | 0.833 | 0.672-0.936 | FALSE | 0.021631992 | 4 | TRUE | -1.97755557 | -4.587168356 | 0 | 1.606472025 | FALSE |
| C1QTNF6 | 0.03571216 | 0.15586772 | 0.87657492 | 0.919872757 | FALSE | 32 | 6 | 30 | 3 | 0.415 | 0.535 | 0.413-0.655 | 0.914 | 0.769-0.982 | 0.167 | 0.064-0.328 | FALSE | 0.013345954 | 4 | TRUE | -2.39225263 | -6.541244881 | 0 | 0.967681396 | FALSE |
| HS6ST2 | -0.06092716 | -0.15466103 | 0.87752272 | 0.919872757 | FALSE | 32 | 10 | 26 | 3 | 0.529 | 0.592 | 0.468-0.707 | 0.914 | 0.769-0.982 | 0.278 | 0.142-0.452 | FALSE | 0.008680297 | 3 | TRUE | -1.91407119 | -5.353887141 | 0 | 2.031811052 | FALSE |
| COMT | 0.044484108 | 0.10782325 | 0.91443679 | 0.942727752 | FALSE | 26 | 17 | 19 | 9 | 0.579 | 0.606 | 0.483-0.72 | 0.743 | 0.567-0.875 | 0.472 | 0.304-0.645 | FALSE | 0.019488535 | 4 | TRUE | -2.73812764 | -1.610099906 | 1 | 2.776671083 | FALSE |
| BMP6 | 0.021678092 | 0.08674317 | 0.93111725 | 0.956107471 | FALSE | 11 | 32 | 4 | 24 | 0.478 | 0.606 | 0.483-0.72 | 0.314 | 0.169-0.493 | 0.889 | 0.739-0.969 | FALSE | 0.005002725 | 3 | TRUE | -2.62992312 | -3.037047956 | 0 | 1.310937041 | FALSE |
| CBLN4 | 0.013253558 | 0.03985552 | 0.96831899 | 0.98321457 | FALSE | 15 | 26 | 10 | 20 | 0.529 | 0.577 | 0.454-0.694 | 0.429 | 0.263-0.606 | 0.722 | 0.548-0.858 | FALSE | 0.010171033 | 3 | TRUE | -6.13014804 | -11.60972024 | 0 | 1.483447871 | FALSE |
| PYDC1 | -0.00602973 | -0.03710713 | 0.97050262 | 0.98321457 | FALSE | 29 | 14 | 22 | 6 | 0.593 | 0.606 | 0.483-0.72 | 0.829 | 0.664-0.934 | 0.389 | 0.231-0.565 | FALSE | 0.014037634 | 3 | TRUE | -0.24289114 | -1.086850306 | 0 | 0.432437263 | FALSE |
| TP53 | -0.002996257 | -0.0289285 | 0.97700192 | 0.986337067 | FALSE | 34 | 5 | 31 | 1 | 0.466 | 0.549 | 0.427-0.668 | 0.971 | 0.851-0.999 | 0.139 | 0.047-0.295 | FALSE | 0.007225036 | 3 | TRUE | -0.64055338 | -2.237191309 | 0 | 0.511676154 | FALSE |
| ATF1 | -0.000258548 | -0.00164097 | 0.99869525 | 0.998695248 | FALSE | 22 | 17 | 19 | 13 | 0.452 | 0.549 | 0.427-0.668 | 0.629 | 0.449-0.785 | 0.472 | 0.304-0.645 | FALSE | 0.013074426 | 3 | TRUE | 1.12227361 | -1.558939868 | 0 | 1.100076719 | FALSE |
