# Supplementary material for: Proteomic Characterization of Grade II Furcation Defects: A Discovery‐Phase Study
Source: J Periodontal Res. 2026 Apr 9;61(8):978–80. doi: 10.1111/jre.70108 (PMC13703785; doi:10.1111/jre.70108)
Supplement: Supplementary file 2 [file JRE-61-978-s003.docx]

**Consent**

All subjects provided written informed consent prior to participation.

**Conflicts of Interest**

The authors declare no conflicts of interest.

**Data Availability Statement**

The data presented in this study are available on request from the corresponding

author due to privacy/ethical restrictions. The data are not

publicly available as they contain information that could compromise

the privacy of research participants. The dataset contains sensitive personal

information, and participant consent did not include public data

sharing.

**Author Contributions**

Georgios S. Chatzopoulos contributed to conception and design, data

acquisition, analysis, interpretation, drafted and critically revised the

manuscript; Dimitra Sakellari contributed to data acquisition, interpretation,

critically revised the manuscript; Eleftherios G. Kaklamanos

contributed to data acquisition, critically revised the manuscript;

Lazaros Tsalikis contributed to conception and design, interpretation,

drafted and critically revised the manuscript. All authors gave their

final approval and agree to be accountable for all aspects of the work.
