# Supplementary material for: Proteomic Characterization of Grade II Furcation Defects: A Discovery‐Phase Study
Source: J Periodontal Res. 2026 Apr 9;61(8):978–80. doi: 10.1111/jre.70108 (PMC13703785; doi:10.1111/jre.70108)
Supplement: Supplementary file 3 [file JRE-61-978-s004.docx]

Table S1. Demographic and Clinical Characteristics of the Study Cohort (N=35).

| Parameter | Value / Frequency |
| --- | --- |
| Demographics |  |
| Age (years) | 54.7 ± 9.1 |
| Sex (Female/Male) | 20 (57.1%) / 15 (42.9%) |
| Smoking (Smokers/Non-smokers) | 16 (45.7%) / 19 (54.3%) |
| Full-Mouth Clinical Parameters |  |
| FMPS (%) | 4.47 ± 3.71 |
| Full-Mouth PPD (mm) | 2.75 ± 0.59 |
| Site-Specific Parameters (Paired) | Test (Furcation) vs. Control |
| Site PPD (mm) | 6.34 ± 1.56 vs. 2.40 ± 0.55 (p<0.001) * |
| Site CAL (mm) | 7.03 ± 1.50 vs. 1.63 ± 0.91 (p<0.001) * |
| Site BOP (%) | 100% vs. 0% (p<0.001) † |

Data for continuous variables are presented as mean ± standard deviation. Data for categorical variables are presented as count (n) and percentage (%).

Data are presented as mean ± standard deviation for continuous variables and as count (n) and percentage (%) for categorical variables. Statistical significance was set at p < 0.05. *P-values were calculated using a paired t-test. †P-value was calculated using McNemar's test.
