# Supplementary material for: Proteomic Characterization of Grade II Furcation Defects: A Discovery‐Phase Study
Source: J Periodontal Res. 2026 Apr 9;61(8):978–80. doi: 10.1111/jre.70108 (PMC13703785; doi:10.1111/jre.70108)
Supplement: Supplementary file 4 [file JRE-61-978-s006.docx]

Supplementary Appendix

1. Supplemental methods
   1.1 Study Population and Inclusion Criteria

Participants were recruited from the Department of Preventive Dentistry, Periodontology, and Implant Biology at Aristotle University of Thessaloniki between October 2022 and June 2025. The study protocol was approved by the Ethics Committee (Protocol No. 155/14-03-2022).

Participants were required to meet the following criteria:

- Be at least 18 years of age with a diagnosis of Stage III or IV periodontitis.
- Have at least one mandibular first or second molar with a Class II furcation defect, a PPD > 4 mm, and a horizontal probing depth ≥ 3 mm.
- Completion of Step 1 periodontal therapy (biofilm control/scaling) prior to sampling.
- The test tooth had to be vital or have received successful endodontic treatment.
- Gingival recession at the furcation site ≤ 2 mm.
- Demonstrate proper oral hygiene, defined as a full-mouth plaque score (FMPS) ≤ 20%.
- Be systemically healthy, with no conditions known to affect periodontal treatment outcomes (e.g., diabetes mellitus).
- Provide signed, written informed consent.

The following conditions resulted in exclusion from the study:

- Pregnancy or lactation (self-reported).
- Presence of any acute infection.
- Requirement for antibiotic premedication.
- Use of antibiotics within the previous 3 months.
- Known allergy to any materials or medications used in the study.
- Teeth with improper endodontic therapy showing clinical signs of infection or teeth with subgingival restorations.
- Tooth mobility of Grade II or III.
- History of radiation therapy to the head and neck region.
- Chronic use of medications known to alter the response of periodontal tissues.
- Smoking ≥ 10 cigarettes per day.

1.2 Clinical Examination

A single, calibrated examiner (G.S.C.) performed all full-mouth clinical examinations at six sites per tooth, excluding third molars. The following clinical parameters were recorded: full-mouth plaque score (FMPS), probing pocket depth (PPD), clinical attachment loss (CAL), gingival recession (REC), and bleeding on probing (BOP). Measurements were made using a University of North Carolina (UNC-PCP15) periodontal probe. Furcation involvement was assessed using both a UNC-15 probe and a Nabers probe (Hu-Friedy; Chicago, IL, USA).

1.3 Sample Collection

GCF was collected at baseline, prior to any periodontal probing. Samples were obtained from one periodontitis site (Grade II furcation, PPD > 4 mm) and one control site (PPD < 4 mm, no BOP) in each patient. After removing supragingival biofilm, the sites were isolated with cotton rolls. A sterile paper strip (Periopaper; OraFlow, Inc., Amityville, NY, USA) was inserted into the crevice or pocket until mild resistance was felt and left in place for 30 seconds. Each paper strip was immediately placed in a sterile Eppendorf^®^ tube and stored at -80°C until analysis. All samples were collected by a single examiner (G.S.C.).

1.4 Proteomic Analysis

Proteomic quantification was performed using the Olink® Reveal platform, which measures 1033 proteins per sample. Sample eluates were prepared according to a randomized plate design generated by Olink’s algorithm based on sample type, patient age, and patient sex. Olink Reveal analysis was conducted following the manufacturer's laboratory instructions.

Next-Generation Sequencing (NGS) was performed on an Illumina NextSeq™ 2000 instrument using the NextSeq™ 2000 P4 XLEAP-SBS™ Reagent Kit (50 Cycles). The platform incorporates extensive quality control (QC) measures, including internal controls spiked into each sample and external controls run on each plate to monitor technical performance and inter-plate variation. Normalized Protein eXpression (NPX) results were generated using NPX Map version 1.3.0 with plate control normalization.

1.5 Bioinformatics Analysis

A bioinformatics pipeline was designed for biomarker discovery using the R programming language in the RStudio environment. The pipeline included exploratory analysis, biomarker discovery, biomarker prioritization, and evaluation.

Exploratory and Statistical Analysis: Principal Component Analysis (PCA) was performed on the NPX values for dimensionality reduction. Analysis of Variance (ANOVA) implemented to test the association of covariates (e.g., sample type, CAL, PPD, smoking, age, gender) with the first two principal components. P-values were corrected for multiple comparisons using the Benjamini-Hochberg method, with an adjusted p-value < 0.05 considered significant (Supplemental Figure S2).

1.6 Biomarker Discovery and Machine Learning:

- Differential Expression: Differential protein expression analysis was conducted between groups, employing a paired statistical test where appropriate. Proteins with an absolute Log₂ Fold Change (|Log₂FC|) > 1 and an adjusted p-value < 0.05 were considered Differentially Expressed Proteins (DEPs).
- Classification Models: Machine learning models were developed to classify samples as 'periodontitis' or control. Generalized linear models (GLMs) and penalized logistic regression (Elastic Net) were implemented. Model training and testing were performed using a 5-fold cross-validation (CV) approach.
- Performance Evaluation: Model performance was assessed by calculating the area under the receiver operating characteristic curve (ROC AUC), sensitivity, specificity, and accuracy, with periodontitis samples treated as true positives. Single-protein ROC analyses were conducted to identify individual biomarkers with an AUC ≥ 0.7. For the multi-protein panel, proteins selected by the Elastic Net algorithm in at least 3 of the 5 CV folds were identified as candidate biomarkers.

1.7 Biomarker Prioritization and Evaluation

To enhance robustness, candidate biomarkers were filtered based on two criteria: 1) NPX values must be greater than the LOD in at least 90% of samples, and 2) the absolute Log₂FC between groups must be greater than the interquartile range (IQR) of the protein's NPX values in the control group. The prioritized biomarkers were used to construct a final Elastic Net model to evaluate their collective classification performance.

1.8 Power Analysis

A post-hoc power analysis was conducted to quantify the probability of detecting the observed effect for each protein. Cohen’s d was calculated, and a two-sample, two-sided t-test was performed with a significance level (α) of 0.05. This analysis was unadjusted for multiple comparisons.

2. Supplementary Tables

Table S1. Demographic and Clinical Characteristics of the Study Cohort (N=35).

| Parameter | Value / Frequency |
| --- | --- |
| Demographics |  |
| Age (years) | 54.7 ± 9.1 |
| Sex (Female/Male) | 20 (57.1%) / 15 (42.9%) |
| Smoking (Smokers/Non-smokers) | 16 (45.7%) / 19 (54.3%) |
| Full-Mouth Clinical Parameters |  |
| FMPS (%) | 4.47 ± 3.71 |
| Full-Mouth PPD (mm) | 2.75 ± 0.59 |
| Site-Specific Parameters (Paired) | Test (Furcation) vs. Control |
| Site PPD (mm) | 6.34 ± 1.56 vs. 2.40 ± 0.55 (p<0.001) * |
| Site CAL (mm) | 7.03 ± 1.50 vs. 1.63 ± 0.91 (p<0.001) * |
| Site BOP (%) | 100% vs. 0% (p<0.001) † |

Data for continuous variables are presented as mean ± standard deviation. Data for categorical variables are presented as count (n) and percentage (%).

Data are presented as mean ± standard deviation for continuous variables and as count (n) and percentage (%) for categorical variables. Statistical significance was set at p < 0.05. *P-values were calculated using a paired t-test. †P-value was calculated using McNemar's test.

Supplementary Table S1: Demographic and Clinical Characteristics of the Study Cohort (N=35).

Supplementary Table S2: Protein biomarkers in Gingival Crevicular Fluid (GCF) - Total candidate biomarkers

Supplementary Figure S1: Biomarkers feature importance from the final machine learning model in periodontitis vs control prediction.

Supplementary Figure S2: Visualization of Principal Component Analysis (PCA) results. Samples are depicted in different colors and shapes based on sample type.
